# The Structural Basis of Gas-Responsive Transcription by the Human Nuclear Hormone Receptor REV-ERBβ

**DOI:** 10.1371/journal.pbio.1000043

**Published:** 2009-02-24

**Authors:** Keith I Pardee, Xiaohui Xu, Jeff Reinking, Anja Schuetz, Aiping Dong, Suya Liu, Rongguang Zhang, Jens Tiefenbach, Gilles Lajoie, Alexander N Plotnikov, Alexey Botchkarev, Henry M Krause, Aled Edwards

**Affiliations:** 1 Banting and Best Department of Medical Research, The Department of Molecular Genetics, University of Toronto, Toronto, Canada; 2 Terrence Donnelly Centre for Cellular & Biomolecular Research, University of Toronto, Toronto, Canada; 3 Midwest Center for Structural Genomics, University of Toronto, Toronto, Canada; 4 Structural Genomics Consortium, University of Toronto, Toronto, Canada; 5 Department of Biochemistry, University of Western Ontario, London, Ontario, Canada; 6 Midwest Center for Structural Genomics, Argonne National Lab, Argonne, Illinois, United States of America; 7 Department of Biology, State University of New York at New Paltz, New Paltz, New York, United States of America; University of Geneva, Switzerland

## Abstract

Heme is a ligand for the human nuclear receptors (NR) REV-ERBα and REV-ERBβ, which are transcriptional repressors that play important roles in circadian rhythm, lipid and glucose metabolism, and diseases such as diabetes, atherosclerosis, inflammation, and cancer. Here we show that transcription repression mediated by heme-bound REV-ERBs is reversed by the addition of nitric oxide (NO), and that the heme and NO effects are mediated by the C-terminal ligand-binding domain (LBD). A 1.9 Å crystal structure of the REV-ERBβ LBD, in complex with the oxidized Fe(III) form of heme, shows that heme binds in a prototypical NR ligand-binding pocket, where the heme iron is coordinately bound by histidine 568 and cysteine 384. Under reducing conditions, spectroscopic studies of the heme-REV-ERBβ complex reveal that the Fe(II) form of the LBD transitions between penta-coordinated and hexa-coordinated structural states, neither of which possess the Cys384 bond observed in the oxidized state. In addition, the Fe(II) LBD is also able to bind either NO or CO, revealing a total of at least six structural states of the protein. The binding of known co-repressors is shown to be highly dependent upon these various liganded states. REV-ERBs are thus highly dynamic receptors that are responsive not only to heme, but also to redox and gas. Taken together, these findings suggest new mechanisms for the systemic coordination of molecular clocks and metabolism. They also raise the possibility for gas-based therapies for the many disorders associated with REV-ERB biological functions.

## Author Summary

Much of human biology, such as sleeping, eating, and even the prevalence of heart attacks, occurs in daily cycles. These cycles are orchestrated by a master “clock” located in the brain. The basic components of this clock are proteins that control the expression of important genes. In this study, we analyze one of these regulatory proteins, named REV-ERB, and show that it is regulated by the combination of heme and nitric oxide gas, both of which are important regulators of human physiology. By determining the 3-D structure of the REV-ERB protein, we were able to uncover clues as to how this regulation occurs. REV-ERB belongs to a protein family called nuclear hormone receptors, which are known to be excellent drug targets. Thus, this paper opens the door to possible gas-based therapies for diseases known to involve REV-ERB, such as diabetes, atherosclerosis, inflammation, and cancer.

## Abstract 3

The heme-regulated nuclear hormone receptor REV-ERB is one of the core transcription factors regulating circadian rhythms. We found that transcription by heme-bound REV-ERB is regulated by NO gas, and a crystal structure of the heme-bound protein uncovers the basis for heme and gas binding.

## Introduction

The closely related REV-ERBα and REV-ERBβ proteins generally act as transcriptional repressors, either on their own, by recruiting co-repressor proteins [1–3], or by competing with the Retinoid-related Orphan Receptors (RORs) α, β, or γ for the same DNA binding sites [4–6]. Physiologically, the REV-ERB proteins play a number of diverse and important roles ranging from the control of circadian biology to the homeostasis of lipids. REV-ERBα and β directly regulate circadian rhythm, both in the brain, and in peripheral tissues, by targeting the circadian clock genes *Bmal1* and *clock* [6–11]. Regulation of lipid metabolism and stimulation of adipogenesis by the REV-ERBs is mediated in part through repression of the apolipoprotein A1 (ApoA1) and apolipoprotein C3 (ApoCIII) gene promoters, which play major roles in cholesterol metabolism [12–14]. REV-ERBs also control inflammatory responses by inducing nuclear factor kappa-light-chain-enhancer of activated B cells (NFκB), interleukin-6 (IL6), and cyclooxygenase2 (COX2) expression, and by repressing IκBα expression [15,16]. In the liver, REV-ERBs also help regulate gluconeogenesis, consistent with an overall role in energy storage and conservation [17].

In *Drosophila*, the REV-ERB homologue, Ecdysone-induced protein 75 (E75) is best known for its role in developmental timing, acting together with the ROR orthologue *Drosophila* Hormone Receptor 3 (DHR3) to control ecdysone-induced molting, pupariation, and eclosion [18]. To perform these functions, E75 appears to require heme as a requisite ligand, bound presumably within its ligand-binding domain (LBD) ligand pocket. E75 is not stable in the absence of heme, nor does the bound heme dissociate readily, suggesting that heme is an obligate component of E75. Interestingly, the presence of heme allows E75 to also bind the diatomic gases nitric oxide (NO) and carbon monoxide (CO), which function to reverse E75-mediated transcription repression [19]. The REV-ERBs also bind heme but, unlike E75, the heme can readily dissociate from the REV-ERBs, and this reversible binding regulates REV-ERB transcription activity [17,20]. The REV-ERB LBDs do not appear to share the ability to bind gases or to respond to redox [20], raising the possibility that E75 and REV-ERBs have evolved two different ways to exploit heme-binding.

The structural basis for heme binding in REV-ERB proteins, and heme and gas binding in E75, is unknown, although mutagenesis and transcription studies have implicated conserved histidine and cysteine residues in heme binding. A crystal structure of the REV-ERBβ LBD in the absence of heme has revealed a classic nuclear receptor (NR) fold, but the mechanisms of heme binding could not be deduced from the structure, because the conserved histidine residue points away from the putative ligand-binding pocket and the conserved cysteine residue was not present in the construct that generated the structure. Moreover, the putative pocket is fully occupied by hydrophobic side chains [21]. The extensive structural similarities between the REV-ERB and E75 LBDs coupled with the apparent mechanistic differences prompted us to explore more deeply the basis for both heme binding and the potential for gas regulation.

The potential involvement of gases in circadian rhythm has been noted in physiological studies for some time, but the molecular mechanisms only began to emerge with the finding that Neuronal PAS domain protein (NPAS2), a CLOCK protein analog, is a hemoprotein [22–24]. NPAS2 and CLOCK heterodimerize with Brain and Muscle Arnt-like protein-1 (BMAL1) to activate transcription of various genes, including the molecular clock components Period, Cryptochrome, and Rev-erb [25–29]. The binding of CO to the NPAS2-heme complex, in vitro, inhibits its binding to BMAL1 and DNA binding [23]. Using transcription assays from native and model promoters in cells, we report that the REV-ERBs are also gas-binding components of the molecular clock and that gas and redox state modulate the structure and function of the REV-ERB LBDs. Using crystallography and spectroscopy, we determined the structure of the heme-bound REV-ERBβ LBD from which we are able to propose a model for heme and gas binding.

## Results

### Heme Binding to REV-ERB LBD Is Reversible

Overexpression of either of the REV-ERB LBDs in Escherichia coli produces apo forms of the repressors. The heme-bound form is produced only if the culture medium is supplemented with hemin (Figure S1). While REV-ERBs expressed with and without hemin supplementation appear to be equally abundant and soluble (Figure S1), they differ strikingly in color, with the heme-bound form intensely red, and the apo form colorless. Incubation of either purified REV-ERBα or β apo LBD with hemin in solution, results in full heme occupancy within seconds or less (unpublished data), while washing the heme-bound LBDs with buffer lacking heme leads to a much slower release of the bound heme (T_1/2_ ∼13–16 h; Figure S2). Overall a K_d_ of approximately 6 μM was observed (unpublished data). Thus, unlike E75, where heme appears to act as a requisite structural component, in the REV-ERBs it can function potentially as a reversible ligand.

### Heme Is Coordinately Bound to Alternative His and Cys Residues

**Figure 1:**
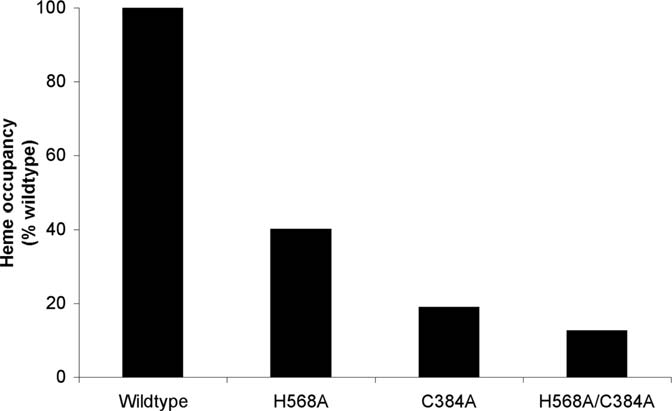
Mutagenesis of REV-ERBβ Heme-Binding Residues As detemined by measurement of construct γ absorption peaks (419 nm), mutation of heme binding residues reduces, but does not abolish heme binding.

Spectroscopic evidence has suggested that the coordination of heme in E75 involves a cysteine residue, and mutagenesis has further suggested that this thiolate bond is contributed by Cys396 (JR, unpublished data) [30], which corresponds to Cys418 in REV-ERBα and Cys384 in REV-ERBβ. Mutagenesis of E75 has also suggested that the second protein-heme coordinate bond is provided by the side chain of His574 (His602 in REV-ERBα; His568 in REV-ERBβ) [19,30]. Consistent with this finding, the mutation of His602 of REV-ERBα (His568 in REV-ERBβ) to phenylalanine essentially eliminates heme binding [17,20]. Our mutagenesis of REV-ERBβ confirms that Cys384 and His568 are both key mediators of heme binding (Figure 1), although the His568 mutant of REV-ERBβ did maintain some heme-binding activity compared with the His602 mutant of REV-ERBα [17,20]. In our REV-ERBβ analysis, the levels of bound heme decrease in each of the purified Cys384 and His568 mutant proteins, but neither of the single mutations to alanine, nor the Cys384A/His568A double mutation, completely eliminate heme binding (Figure 1). These differences may be attributable to the different choices for amino acid substitution (Ala versus Phe), or other differences between the two proteins. Thus, residues in addition to these particular Cys and His residues must also contribute to heme binding.

### Coordinate Bonds Change as a Function of Redox State

The structures of other heme-containing transcription factors are modulated by redox state [23,31–33]. To investigate the effects of redox state on the interaction of REV-ERBs with heme, we subjected the heme-bound proteins to electronic absorption spectral analysis. The heme in coordinately bound proteins absorbs at characteristic wavelengths, producing what are referred to as α, β, and γ (or Soret) heme absorption peaks. The existence, positions, and sizes of these peaks provide insight into the oxidation and spin states of the iron center and the number and types of coordinate bonds formed.

**Figure 2:**
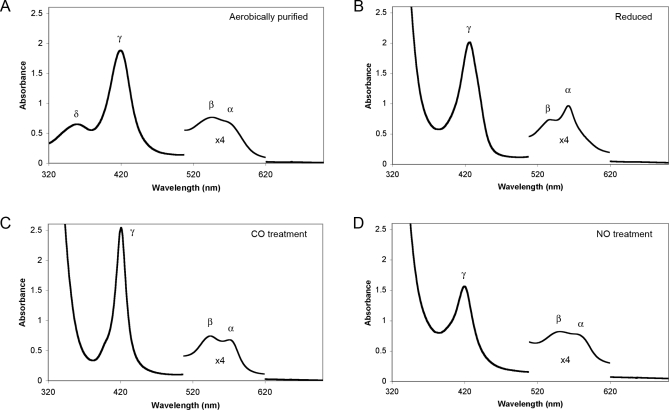
REV-ERBβ Contains Heme and Can Be Reduced and Binds Diatomic Gases In Vitro (A) Electronic absorption spectrum of 0.5 mg/ml recombinant REV-ERBβ aerobically purified from bacteria (supplemented with 50 mg/l hemin during growth). (B) REV-ERBβ plus 2 mM sodium hydrosulphite. (C) Reduced REV-ERBβ plus 100 μM CO. (D) Reduced REV-ERBβ plus 100 μM NO. The region from 500 nm to 620 nm is inset and magnified for presentation of the α and β peaks. The analogous spectra for REV-ERBα were similar.

**Figure 3:**
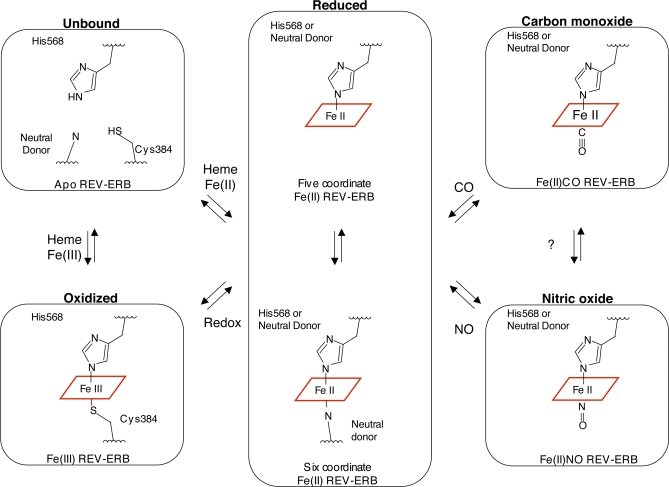
REV-ERBs Can Assume at Least Six Different Heme Binding States In the absence of heme, the ligand pocket of REV-ERBs is stabilized with the side chains of core hydrophobic residues. Heme can bind to the REV-ERBs in either reduced Fe(II) or oxidized Fe(III) states, and while reduced is in a five or six coordinate state. REV-ERBs also bind the diatomic gases CO and NO through the creation of a coordinate bond between the Fe(II) center of heme.

The oxidized Fe(III) REV-ERBβ LBD yielded an absorption spectrum (Figure 2A) that was almost identical to those produced by aerobically purified *Drosophila* E75 [19] and the bacterial thiolate-heme Fe(III)-containing transcription factor CooA [31]. All three proteins exhibit characteristic α (∼575 nm, shoulder), β (542 nm), and γ (419 nm) absorption peaks as well as a prominent δ-band (359 nm); altogether, these are indicative of a hexa-coordinated heme bound to at least one thiolate (e.g., Cys) group [30]. Upon subjecting the REV-ERBβ LBD to the reducing agent sodium hydrosulphite (dithionite), the resultant shifts in absorption peaks (Figure 2B) indicate reduction of the iron center from Fe(III) to Fe(II), and loss of the thiolate coordinate bond (diagrammed in Figure 3); this reduction is seen most readily by the loss of the δ-band (359 nm). We have also reported elsewhere [34], using magnetic circular dichroism and resonance Raman spectroscopy, that reduction of the REV-ERBβ iron center appears to yield both a 5-coordinated system, with a single neutral residue coordinate bond (e.g., His or Pro), as well as a 6-coordinated system with two neutral residue bonds (Figure 3). Thus, the reduced REV-ERB-heme complex comprises at least two structural states in which the heme-coordinating amino acid side chains change. This form of redox-regulated coordinate bond switching is not unique to REV-ERBs. For example, similar side chain-switching states have been observed in CooA (Cys^75^ to His^77^), a bacterial CO-responsive heme thiolate protein transcription factor [35–37], and in NPAS2 (Cys^170^ to His^171^), whose axial coordinate bonds are also different in the Fe(III) and Fe(II) states [23,33,38]. In these transcription factors, these redox-dependent structural changes also result in functional changes for their host proteins. Redox was also shown to modulate E75 coordinate bonding and function [19], but it remains to be seen if the redox-dependent structural changes in the REV-ERBs also have analogous functional consequences.

### REV-ERB-Heme Proteins Can Bind NO and CO Gases

As pointed out earlier, many coordinately bound heme proteins, including proteins such as hemoglobin, cytochromes, and the transcription factors CooA and NPAS2, have the added ability to bind gases [23,31,37]. This ability is also the case for the REV-ERB insect orthologue E75 [19]. As a first step in assessing the gas-binding potential of REV-ERB proteins, the reduced REV-ERBβ LBD was incubated with CO or NO gas and analyzed for characteristic changes in the electronic absorption spectra. The altered electronic absorption profiles confirm direct binding of both diatomic gases to the heme-bound forms of both REV-ERB LBDs. When incubated with either NO or CO, the reduced (but not oxidized) REV-ERBβ LBD exhibits classic shifts in the absorption peaks (Figure 2C, 2D) similar to those seen in other gas-bound hemoproteins [31,39,40]. More detailed studies of the heme in REV-ERB LBDs [34] have shown that the NO and CO gases bind opposite to a neutral side chain in a 6-coordinated state (Figure 3). Thus, the heme-binding data reported previously [17,20] and the spectroscopic data reported here and in Marvin et al. [34] reveal that REV-ERBs comprise modular ligand systems that can adopt a minimum of six different LBD structural states (Figure 3), each of which has the potential for distinct functional interactions, transcriptional outputs, and biological roles.

### NO Reverses REV-ERB Mediated Repression In Vivo

**Figure 4:**
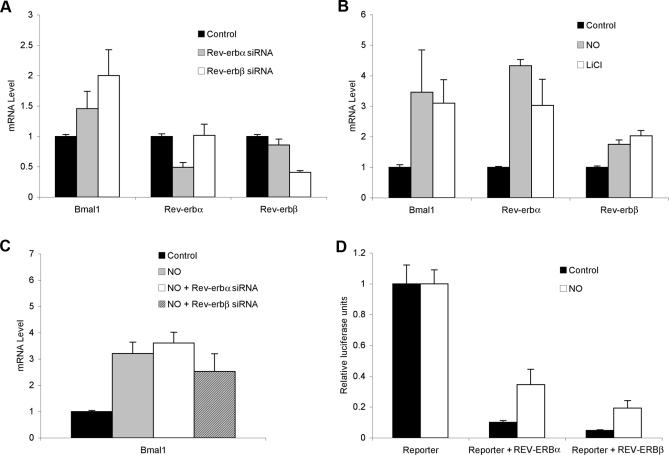
REV-ERBα and β-Mediated Repression Is Relieved by a NO Donor (A) Endogenous mRNA levels of Bmal1 and Rev-erbα and β in control and Rev-erbα and β siRNA treatments. (B) Bmal1 and Rev-erbα and β gene expression following treatment with either 300 μM Deta/NO or 20 mM LiCl. (C) Comparison of Bmal1 gene expression levels in control, Deta/NO- and siRNA-treated cells. (D) Transient transfection of 293T cells with GAL4-REV-ERBα or GAL4-REV-ERBβ results in repression of the UAS_GAL4_-containing luciferase reporter. Treatment of the cells with Deta/NO leads to a 3-fold derepression of luciferase expression. All experiments were performed in triplicate, with the error bars showing sample to sample variation. Experimental data shown are representative of a minimum of three repeats under the same conditions.

Among the many genes targeted for repression by the REV-ERB proteins are their own genes and the clock gene Bmal1. To test the effects of NO or CO on the activities of REV-ERBα and β in vivo we monitored transcription levels of the endogenous Bmal1 and Rev-erb genes in human embryonic kidney (HEK) 293T and hepatocellular carcinoma (HepG2) cells in response to gas. We first confirmed that transcription from these genes is regulated by the REV-ERBs by measuring their transcription in the presence and absence of Rev-erb small interfering RNA (siRNA). Figure 4A shows that each of the Rev-erb siRNAs specifically targets its corresponding Rev-erb gene. As expected, the knock-down of either Rev-erb gene results in an increase in Bmal1 expression (1.5–2-fold; Figure 4A), due presumably to derepression of ROR-mediated transcriptional activation. Effects were readily observed within 12 h of siRNA treatment, and peaked at 72 h post-treatment.

We then asked whether NO, supplied by the chemical donor diethylenetriamine/NO (Deta/NO), relieves REV-ERB-mediated repression of the endogenous Bmal1, Rev-erbα, and Rev-erbβ genes. Addition of Deta/NO increased levels of Bmal1, Rev-erbα, and Rev-erbβ mRNAs by 2–3-fold (Figure 4B). The addition of Li^2+^, which has been shown to cause REV-ERB degradation via inhibition of Glycogen Synthase Kinase-3 β (GSK3β) kinase-mediated phosphorylation [41], led to a similar derepression of the endogenous Rev-erb and Bmal1 target genes. Importantly, combining NO and siRNA treatments did not have additive effects, suggesting that NO acts via derepression of the REV-ERBs and not via a parallel pathway (Fig 4C). NO-dependent transcription was also observed in HepG2 cells, with the exception that NO-mediated upregulation of ROR/Rev-erb target gene expression was only ∼2-fold (Figure S3). This may reflect lower levels of available heme in this cell type, as heme levels vary significantly in different cell types and in other cell states [42,43]. Analogous studies conducted in the presence of 500 ppm CO, yielded only modest changes in REV-ERB target gene expression (unpublished data). This minimal response may be due to differences in affinity or effectiveness between NO and CO in cells, or to differences in the effectiveness of the different gas delivery protocols used (see Methods).

To provide evidence that NO acts as a direct regulator of heme-bound REV-ERB proteins, the LBDs of either REV-ERBα or β were fused to the DNA-binding domain of yeast GAL4, and their activities tested using a luciferase reporter regulated by a thymidine kinase promoter containing upstream activating sequences (UAS)_GAL4_ binding sites. As expected, co-transfection of the GAL4-REV-ERBα or GAL4-REV-ERBβ fusion proteins repressed transcription driven by the UAS-containing thymidine kinase promoter (Figure 4D). This repression was reversed by greater than 3.5-fold by the addition of either of two NO donors, Deta/NO (Figure 4D) or S-nitroso-N-acetyl-l,l-penicillamine (SNAP) (unpublished data), suggesting that the REV-ERBs are direct targets of NO. As earlier, similar studies with 750–2,000 ppm CO had a more modest effect (∼15% of NO effect; unpublished data). In summary, both REV-ERB proteins are transcriptional repressors whose activities can be reversed by NO binding.

### Effects of Gases on REV-ERB Co-Repressor Binding

**Figure 5:**
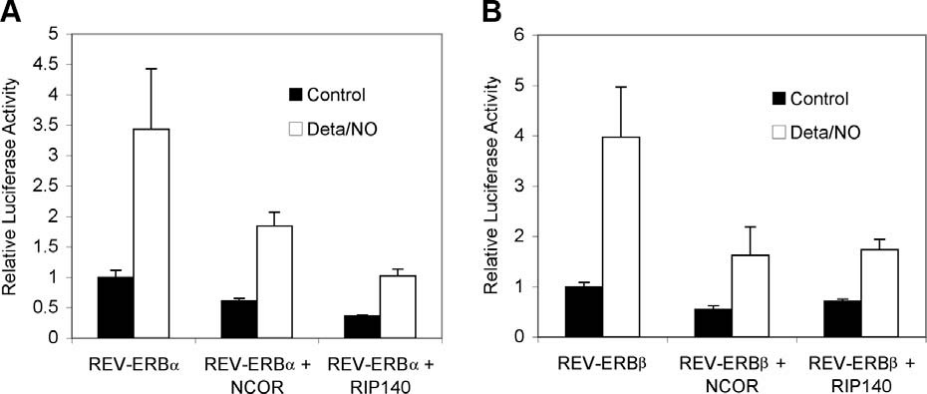
GAL4-REV-ERBα (A) and GAL4-REV-ERBβ (B)-Mediated Repression Is Augmented by Co-transfection with Equimolar Amounts (370 fmol/well) of Full-Length NCOR or RIP140 Co-repressors Repression, in all cases, is significantly relieved by the addition of Deta/NO. The averages from triplicate experiments are presented as relative luciferase activity.

To determine if the heme and gas effects on REV-ERB activity might be attributable to the recruitment of co-repressor proteins, GAL4-REV-ERBα and GAL4-REV-ERBβ fusion proteins were co-transfected with full-length co-repressor expression constructs into 293T cells. As expected, addition of the known REV-ERB co-repressor Nuclear Receptor Co-repressor (NCOR) to GAL4-REV-ERB transfection assays increases repression by 2–3-fold. Similar results were obtained by coexpression with another co-repressor, Receptor Interaction Protein 140 (RIP140), which has not been previously tested for REV-ERB binding (Figure 5A, 5B). This augmented NCOR or RIP140-mediated repression is reversed by the addition of Deta/NO (Figure 5A, 5B). Similar reductions in Gal4-REV-ERB/co-repressor mediated repression were obtained by treating the transfected cells with valproic acid, which is an inhibitor of the histone deacetylases that are recruited by NCOR and RIP140 [44–46]. We conclude that NO signaling reduces REV-ERB repression activity, at least in part, by overcoming the recruitment or activities of these co-repressors.

**Figure 6:**
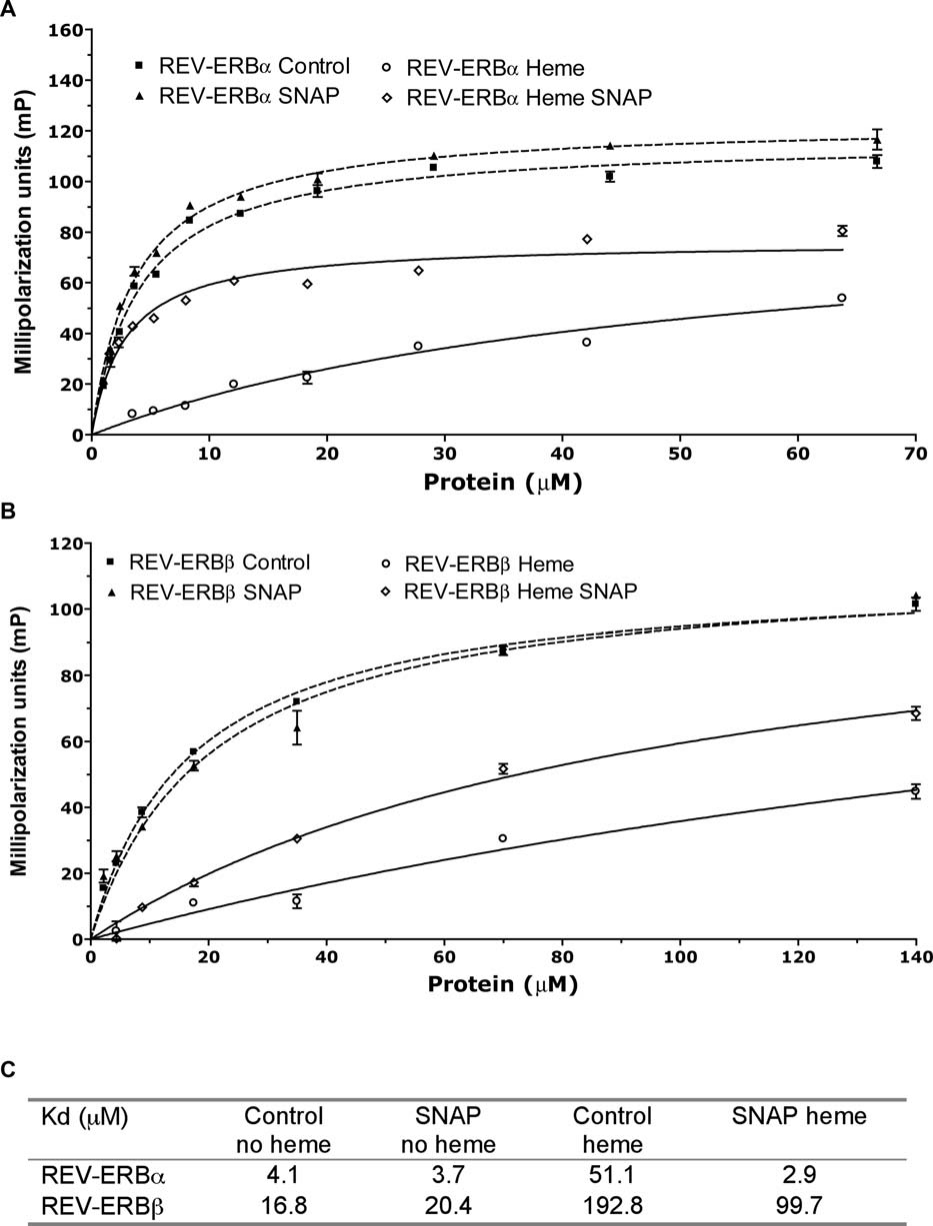
REV-ERB Binding of NCOR Peptide Is Dependent on Heme and NO Ligands Fluorescence polarization titration curves for the binding of 110 nM NCOR peptide to apo (dashed line) or heme-bound (solid line) REV-ERBα (A) and β (B) LBDs. Greater mP values indicate increased binding of the peptide to REV-ERBs. As expected, the nature of REV-ERB LBD binding of NCOR peptide is dependent on heme and in the heme-bound state are responsive to NO gas. (C) Calculated K_d_ values.

The effects of heme-binding on REV-ERB function are unclear. Previous studies have shown that the availability of heme negatively affects the ability of REV-ERB proteins to bind co-repressor peptides in vitro but is required for the REV-ERB proteins to interact functionally with co-repressors in vivo [17,20]. To explain this finding, it has been suggested that co-repressor interactions in vivo must be modulated by interactions or conditions that are not reflected in experiments with purified components. To test if the in vitro interactions could be influenced by gas, we used fluorescence polarization to follow the recruitment of peptides corresponding to the LXXI/HIXXXI/L interaction domain I (IDI) of NCOR (Figure 6) and Silencing Mediator for Retinoid and Thyroid hormone receptor (SMRT) (unpublished data) in the absence and presence of heme and gas. As expected, based on the previous study, both peptides interact specifically with the REV-ERBα and β LBDs (Figure 6), and the addition of heme acts negatively on co-repressor peptide binding. As might also be expected, only the heme-bound form of the REV-ERB LBDs are responsive to the addition of NO gas (Figure 6A–6C and unpublished data). As with heme binding though, this effect is the opposite of that which occurs in vivo, with NO acting to increase co-repressor peptide recruitment, rather than blocking it. We can only conclude, as did Yin et al. [17], that interactions or conditions that exist in the cell, are not reflected in the in vitro system.

### Structural Analysis of REV-ERBβ Bound with Heme

To shed light on the structural basis of heme, gas, and redox regulation, we crystallized the REV-ERBβ LBD in the heme-bound state. The formation of well-ordered crystals required the addition of trypsin to the crystallization solution [47]. Two identical structures were obtained using constructs comprising either the complete LBD (residues 212–579) or the LBD with an internal deletion (residues 241–579 Δ 275–357). Both 1.9 Å resolution structures include α-helices 3–11, (residues 381–576; REV-ERBβ_381–576_), which is slightly larger than the fragment used to derive the unliganded LBD structure [21]. Both of the crystals were obtained under nonreducing conditions.

**Figure 7:**
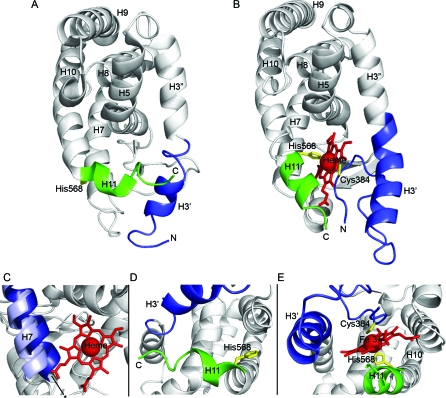
The Structure of REV-ERBβ LBD in Complex with Fe(III) Heme (A) Structure of REV-ERBβ LBD without [21] and with (B) heme bound in the ligand-binding pocket. (C) The position of H7 in the apo- (dark blue [21]) and heme-bound (light blue) states of REV-ERBβ. Detailed view of heme-binding site of REV-ERBβ LBD in the absence (D) [21] and presence (E) of heme. The core of the protein is in gray, N-terminal part of H3 (H3') is in blue and H11 is in green. The side chains of Cys384 and His568 are shown in yellow and heme is colored red.

The two REV-ERBβ_381–576_ Fe(III) heme structures verify that the heme-binding pocket is in fact present at the same position as ligand-binding pockets observed in other NR family members (Figure 7B). As predicted by the mutagenesis and spectroscopic analyses for the oxidized state of REV-ERB, a single heme molecule is hexa-coordinated within the pocket by Cys384 and His568 side chains. As mentioned above, Cys384 was not included in the previously published unliganded receptor structure constructs [21].

The structural changes that facilitate heme binding are confined primarily to helices 3, 7, and 11. In the absence of heme, helix 3 breaks at Pro411 allowing its N-terminal portion to move into the unliganded pocket (Figure 7A) [21]. A number of aromatic residues from this helix face into the pocket, contributing substantially to the hydrophobic core that stabilizes the unliganded structure. In the presence of heme, helix 3 straightens, swinging the end of its N-terminal half (Cα atom Gly398) 16.4 Å away from its position in the unliganded structure (Figure 7B). Although helix 7 shifts in a less dramatic manner, the 3.0-Å movement (Cα atom Leu482) further increases the pocket volume. The movement of residues 480–483, in particular, allows heme and its propionate side chains to assume their observed planar orientation (Figure 7C). In the absence of heme, helix 11 shields the hydrophobic core by bridging helices 10 and 3. To facilitate heme binding, it also undergoes a major conformational change, swinging its C terminus (Cα atom Leu576) 15 Å away from the ligand-binding pocket, forming a gently curving, uninterrupted α-helix that covers the ligand pocket (Figure 7A, 7B).

The unprecedented formation of LBD-ligand coordinate bonds involves some equally novel and elegant structural changes. First, the imidazole side chain of His568 makes a ∼120° rotation around the axis of the helix to allow bonding with the Fe(III) heme center (Figure 7D, 7E). Cys384, the other coordinate bond-forming residue in this Fe(III) structure, derives from a flexible loop N-terminal to helix 3, which does not appear in the apo structure (Figure 7D, 7E). In addition to opening the pocket and correctly positioning the two heme-coordinating residues, the newly positioned helices and loop also help to shield the hydrophobic heme moiety from the solvent, with only 8% (66 Å^2^) of the ligand exposed. This value falls well within the expected normal range of 1%–28% for hemoproteins [48].

**Figure 8:**
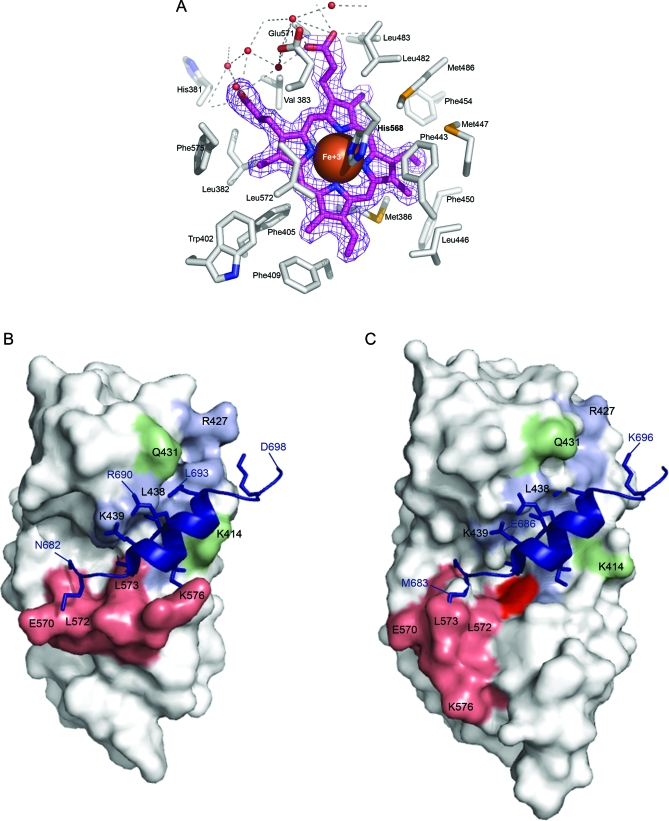
Effects of Heme Binding on the REV-ERBβ Ligand-Binding Pocket and Co-Repressor Binding Groove (A) A detailed view of the heme-bound REV-ERBβ ligand-binding pocket. Electron density of the porphyrin ring and iron is closely bounded by hydrophobic sidechains of the ligand-binding pocket. The hydrophilic propionate groups of heme interact with water molecules (red spheres) near the surface of the protein. Model of Apo-(B) [21] and heme-bound (C) REV-ERBβ LBD structures in complex with a SMRT ID-I co-repressor peptide [101]. Peptide residues (N682–D698) are in dark blue, groove residues previously identified as being important in peptide binding are in blue (F409, V417, K421, R427, V435, L438, K439) [21,59,60], residues in H11 are in salmon (which also include residues previously identified as important to co-repressor binding; L572, F575, K576), heme is in red, and other residues in the groove that shift upon heme binding are in green (K414, Q431).

The majority of residues surrounding heme in the pocket stabilize heme binding via van der Waal interactions. Within 4 Å of the heme moiety are 25 residues (Table S1) derived from five different regions of the REV-ERBβ secondary structure (H3, H5, H7, and H11, and loop N-terminal to H3). The majority of these residues form the core of the apo-structure [21], and must swing out and away to facilitate heme binding (Figure 8A). In other hemoproteins the residues forming hydrophobic heme contacts include Ile, Leu, Val, Phe, Trp, and Tyr [48]. The REV-ERBβ pocket is also enriched with these residues, along with six phenlyalanines (Table S1). With two exceptions, all of these residues are conserved in REV-ERBα and among all the vertebrate REV-ERBβ orthologues. Although these contacts occur all around the heme ligand, Trp402, Phe405, and Phe454 are striking examples of how van der Waal radii of the protein side chains and heme can interlock (Figure 8A). Taken together, these precisely fitted hydrophobic contacts must contribute significantly to the strength and specificity of heme binding.

Aside from Cys384 and His568, the only other polar residues within 4 Å of heme are His381 and Glu571, and while at this point their role is undetermined, their presence in an otherwise nonpolar environment, and their conservation in other REV-ERBβ homologues, suggests a functional role (Figure 8A, Table S1). Ligand -binding specificity in many NR ligand-binding pockets often involves hydrogen bonding between polar group(s) on the ligand and charged residue(s) of the LBD. The most common polar interaction in the NR pocket is with an arginine side chain that precisely orients ligands to ensure specificity [49]. While this Arg is conserved in the REV-ERB LBDs, neither it nor any other Arg residue faces the ligand-binding pocket in the apo or liganded forms [21]. Glu571, however, is positioned 3.8 Å from the negatively charged propionate groups of heme (Figure 8A). This is unusual because the carboxy termini of heme propionate groups usually interact with positively charged residues such as Arg or Lys [48]. It may be possible that the negatively charged propionate side chains are repelled by the acid group of Glu571 in a way that helps to properly center the heme group, or perhaps helps to facilitate exchange. Interestingly, in REV-ERBα and E75 the analogous residue is lysine, which would be predicted to attract the carboxy termini of the heme molecule, as observed in other heme-binding proteins.

The other polar residue within close proximity to the heme group is His381, which is close to the heme coordinating Cys384 residue, and is highly conserved throughout vertebrate REV-ERBβ homologues (Figure 8A). Given the spectroscopic data, which suggest switching of coordinate bonds from a Cys to a neutral residue such as histidine upon heme reduction, His381 is a good candidate for this substituting residue. Indeed coordinate bond switching in other heme proteins tends to involve nearby residues [33,35]. Interestingly, within this loop there are three other His residues that may also be capable of coordinate bond formation. All three are also within HXXC motifs (Figure S4), which serve as metal binding sites in the unstructured loops of olfactory receptors [50] and other hemoproteins. Alternative switching between these Cys/His residues has the potential to ratchet the loop peptide along the plane of the heme molecule, and to reshape the external LBD surface into novel protein interaction sites.

Also worth noting is that the residue next to the coordinately bound Cys384 is a proline (Pro385). This highly conserved Cys-Pro duo fits a consensus for “heme regulatory motifs,” which also include flanking residues such as His, Leu, Val, Met, Lys, Arg, and Asp [51–53]. This heme regulatory motif in REV-ERBβ includes six of those seven residues (Figure S4). Such motifs have been shown to be capable of binding heme reversibly with low micromolar affinity. Mutational analyses of the corresponding prolines in other heme thiolate proteins suggest that these residues help to direct the Cys residue toward the heme moiety, as well as to contribute to the reversibility of Cys-heme binding [54–56]. The *Drosophila* E75 LBD is a notable exception to this reversibility, although this may be explained by the presence of a second heme binding cysteine (Cys468) that is not flanked by a proline and has no counterpart in the REV-ERBs [19,30].

A final consideration based on this structure is how the NCOR and SMRT co-repressor peptide-binding site on the LBD surface changes upon the addition of heme. Heme binding appears to affect the previously characterized co-repressor binding site in two ways. First, the hydrophobic groove becomes broader. Second, helix 11, at the base of the groove, swings away from the binding site. This ligand-dependent movement of H11 from the co-repressor binding site supports the notion that H11 serves as a proxy for the missing H12, which in other NRs would serve as a platform for co-repressor binding [21]. Both of these heme-induced changes are predicted to impact negatively on co-repressor binding. It is interesting to note that the helices that show the greatest movement upon heme binding are those that border the co-repressor binding groove H3–5, H10, and H11 (Figures 7 and 8) [57–60].

A number of specific REV-ERBβ residues are critical for co-repressor binding, and have been identified previously [60]. Examples include residues from H11, which are in position to form a number of critical co-repressor contacts in the apo form (L572, F575, K576) but that are shifted dramatically in position by movement of the helix, making them unlikely to maintain these interactions (Figure 8B, 8C). Likewise in H3, F409, which has also been identified as essential [60], shifts from presumably holding H11 in position for co-repressor interaction to becoming a hydrophobic contact for heme. K414 of H3 also appears to make a critical shift that leads to widening of the hydrophobic peptide-binding groove. At either end of the hydrophobic groove, there are also charged residues (K421, R427, and E570) that have been predicted by modeling to play important roles in anchoring the NCOR peptide [21]. Two of these three residues, R427 and E570, shift dramatically away from the co-repressor binding groove in the heme-bound form (Figure 8B, 8C) [21]. Notably, hydrophobic vinyl and methyl groups from the heme moiety also extend to the surface of the groove close to the region where H11 was positioned. While this does not appear to provide interference, it does indicate the possibility for heme to either interact or interfere with co-repressor binding under different conditions.

These alterations in the co-repressor binding site are consistent with the effects of heme on peptide binding in vitro. Presumably, disruption of one of the coordinating heme ligands by NO would restore peptide binding by relieving the strain imposed on the LBD by the hexa-coordination of heme. Changes to the structure of the binding site cannot, however, explain why heme and the presence of NO have the opposite effects in vivo. The answer to this apparent paradox will most likely require structural analyses under different conditions, in the presence of other REV-ERB or co-repressor protein domains, or with other known or unknown cofactors.

## Discussion

### A New Heme Binding Fold

Over 20 different protein folds can specifically bind b-type heme, which is the most abundant of the hemes and serves as the functional group for essential proteins such as hemoglobin, myoglobin, and cytochrome b5. Under different evolutionary constraints and pressures, these various heme-binding folds have adopted additional functional properties, which include electron transfer, redox sensing, and the sensing or transport of various gases [48]. The REV-ERBβ_381–576_/heme structure adds a new and highly dynamic representative to the heme binding-fold family.

The molecular volume of heme (∼520 Å^3^) is relatively large in comparison to most other NR ligands. Hence, the conformational changes that allow entry and occupancy of the apo LBD pocket are considerable. Such structural plasticity has been observed for an increasing number of NRs (e.g., Ecdysone Receptor [ECR] [61], Liver X Receptor [LXR] [62], and Estrogen Receptor [ER] [63]). This plasticity is an important point, as it indicates the potential for other “orphan” receptors, with seemingly inadequate ligand-binding pockets, and “constitutive” activities, to also be regulated by novel small molecule ligands within their various natural in vivo environments.

### A Multifunction, Multipurpose Ligand System

As with many other heme-containing proteins, which include E75 [19], both REV-ERB proteins are also able to monitor redox state and to bind gases. E75 and the REV-ERBs are unusual however, in that while discriminating against O_2_, they are able to bind both NO and CO gases in vitro. Although the CO gas responses observed in vivo were much weaker than those observed for NO, this may be a consequence of the different methods of gas delivery used, or differences in the cellular functions and biochemistry of the two gases.

The different kinetics of gas and heme binding to the REV-ERB LBDs, and the different rates at which these molecules are produced and metabolized within the body, suggest that these ligands may have different physiological roles in different tissues. Gas and redox exchange observed in vitro occurs within seconds, whereas heme exchange requires many hours. In the body, changes in redox and gas levels can be rapid [32,64], whereas heme levels oscillate over hours or days [42,43]. It may also be of relevance that heme exchange does not appear to be possible for the fly orthologue E75, such that the levels of E75 accumulation in the cell are dependent on the abundance of available heme [19]. Thus, while both E75 and REV-ERB proteins may function as heme sensors, REV-ERBs appear to have the added ability to function in the absence of heme.

Although we also attempted to capture the structure of REV-ERBβ in reduced Fe(II) and gas-bound states, and were able to derive crystals, the latter diffracted poorly due possibly to the predicted multiplicity of Fe(II) coordinate bond isoforms (Figure 3). This heterogeneity would be consistent with our spectroscopic analyses, and those of Marvin et al. [34], which suggest that the Cys384-heme coordinate bond is replaced in the Fe(II) population by one of several alternative neutral donors. It is tempting to speculate that His381, which is conveniently positioned just N-terminal to Cys384, may serve as one of these residues. In fact, the ∼133 residue loop between helices 1 and 3 (Figure S4) contains at least 23 residues that could coordinate heme (nine His residues, seven Met residues, and seven Cys residues). This abundance of His, Met, and Cys residues is around three times their general frequency in the human proteome. There are also three more histidine residues (His395, His399, and His475) surrounding the ligand-binding pocket that could serve as alternate binding partners. If any of these residues do in fact form alternative coordinate bonds, this would lead to an additional and unprecedented number of LBD conformational and functional variants.

### Effects of Heme and Gases on Co-Repressor Binding

In terms of how heme and gases affect REV-ERB LBD functions, our results suggest a major role for both ligands in co-repressor recruitment. The presence of heme leads to significant broadening of the co-repressor-binding groove and a highly unfavorable redistribution of interacting residues, consistent with the dramatic drop in co-repressor peptide binding observed in vitro. Addition of NO to the heme-bound LBD reverses the negative effect of heme on peptide binding, suggesting that NO acts by increasing the affinity for co-repressor binding. As with heme though, the effect of NO gas on REV-ERB activity in cultured cells appears opposite, with, the addition of gas leading to a drop in the ability of REV-ERB proteins to repress transcription. This apparent dichotomy in the effects of heme and gas on co-repressor function in vitro and in vivo cannot yet be explained by current structural findings. Structures for reduced and gas-bound forms of the LBD may help solve this apparent paradox. On the other hand, the answer may involve structures of additional parts of the REV-ERB or co-repressor proteins, or other interacting factors.

While NCOR is an established co-repressor for the REV-ERBs [65], this study is the first to implicate a role for RIP140 as a REV-ERB co-repressor. RIP140 contains ten known NR interaction motifs, which provide the functional capacity to interact with different classes of NR partners [66]. Consistent with a role in REV-ERB modulation, RIP140 has also been implicated in the regulation of lipid and glucose metabolism [67]. Furthermore, like the REV-ERBs, its expression is also induced by retinoic acid [68,69], and it is an important regulator of skeletal muscle metabolism [70,71]. Hence, this interaction may have important implications for the study of cancer and metabolic diseases.

### Physiological Roles Implicated by REV-ERB Functions and Ligands

The evolution of a central circadian clock has allowed higher eukaryotes to anticipate the daily light and dark cycles, and to coordinate these with appropriate changes in behavior and metabolism [72]. As components of the molecular clock, the REV-ERBs and RORs play important roles in this daily cycling by regulating clock gene expression in a ligand-dependent manner. They also play a central role in the regulation of glucose and lipid metabolism within metabolically intensive tissues [17,73,74]. We propose that the REV-ERBs coordinate these two different functions by monitoring the metabolic indicators/signals: heme, NO, and/or CO and redox.

Heme has long been recognized as an important molecule in metabolism. It is required for oxygen and carbon dioxide transport, for cytochrome function in the mitochondria and for the neutralization of reactive oxygen species arising as a consequence of metabolism. It is also a required component of the cytochrome P450s that produce and break down most lipids, including those that serve as the ligands of most NRs [23,42,43,48,75–77]. More recently, heme has been shown to oscillate during the circadian cycle, to influence the circadian cycle, and to be a component of the circadian clock proteins Period 2 (mammalian PER 2 [mPER2]), NPAS2, and now the REV-ERBs [23,42,43,76,78]. Given that heme is so central to respiration and other central metabolic processes, and that its abundance appears to oscillate over time, we suggest that heme serves as a fundamental measure of the diurnal metabolic state and as such provides feedback through the REV-ERBs, and other clock proteins, to entrain the molecular clock.

Further support for this central role of heme is the reciprocal nature of heme and CO production. Expression of Aminolevulinate synthase 1 (ALAS1), the rate-limiting enzyme in heme synthesis, is positively regulated by the clock complex mPER2/NPAS2/BMAL1, making this expression circadian in nature. As heme abundance increases, so does the expression of the heme-regulated enzyme Heme Oxygenase and its product CO. In turn, the presence of CO leads to dissociation of the mPER2/NPAS2/BMAL1 complex and down-regulation of *Alas1* transcription. Consequently, heme concentrations fall, and the cycle is reset [8,23,42,79]. As REV-ERBs also bind heme, their expression is heme dependent and they repress *Bmal1* [6,11,17,20,76], thereby forming a second reciprocal feed-back loop between heme synthesis and circadian rhythm.

Heme is also an essential component of the NO and CO producing enzymes Nitric Oxide Synthetase and Heme Oxygenase. Not surprisingly, both NO and CO production have also been shown to oscillate diurnally. In the suprachiasmatic nucleus of the hypothalamus, where the central molecular clock is located, the activity and products of these enzymes peak during the night [77,80,81]. Given that the transcriptional activities of NPAS2 [23], REV-ERBα and β have all now been shown to be gas responsive, these diatomic gases may provide a secondary layer of regulation to the heme-enriched molecular clock. The membrane permeability and short half lives of these gases make them ideal neurotransmitters [82,83] for communication between the different nuclear regions of the hypothalamus, where circadian and metabolic homeostasis are regulated.

The cycling of redox state offers a third potential mechanism for entrainment of the molecular clock. Redox homeostasis can be affected by the generation of reactive oxidant species (ROS), a large proportion of which arise not surprisingly from mitochondrial respiration. The redox state of a cell, or organelles, is dependent on the ratio of ROS generated by metabolic activity and the abundance of antioxidants, both of which cycle diurnally (reviewed in [84–86]). Aside from the damage that ROS can cause, these molecules have become recognized as important signaling molecules. Interestingly, ROS signaling is commonly associated with stress response [87], and the hypothalamus controls the body's response to stress [88,89]. Considering the redox-sensing abilities of mPER2 [76], NPAS2 [90], and the REV-ERBs, it seems likely that both central and peripheral molecular clocks are also entrained by redox signaling. As means of entraining circadian rhythm, redox cycling is not without precedent. It can be traced back to the primordial biological clock of cyanobacteria, where light and the redox state, as a measure of metabolism, synchronize the global transcriptional rhythm of the organism [91].

In summary, our findings indicate a complex reciprocal relationship between metabolism and the molecular clock in which the molecular clock serves to synchronize circadian metabolic activity [14,17,20,23,33,42,57,73,76,79], and in turn heme, diatomic gases, and redox serve as local and systemic indicators of this activity, thereby helping to entrain clocks within different tissues. Thus, the circadian cycle is not only a means of metabolic regulation, but is in fact a metabolic cycle [85].

### REV-ERBs in Disease and Treatment

As a whole, the combined ligand set of heme, gases, and redox state, combined with the even greater number of induced structural changes in REV-ERB LBDs, provide the potential for many different protein interactions and physiological functions that are in line with the central role that the REV-ERB and E75 proteins serve in coordinating metabolic processes with circadian and developmentally timed events. Taken further, the rapid responses of REV-ERB proteins to gas signaling, and the importance of these gas- and REV-ERB-regulated physiological processes, illustrate the potential for novel, gas-based therapies for the treatment of related diseases such as mood and sleep-based disorders, depression, obesity, diabetes, atherosclerosis, and osteoarthritis.

## Methods

### Expression constructs.

For bacterial expression of the LBD of REV-ERBα (GenBank [http://www.ncbi.nlm.nih.gov/Genbank] accession: CAB53540), the construct comprised residues 274–614 with an internal deletion of residues 324–422. The first REV-ERBβ (GenBank accession: CAG33715) construct for bacterial expression comprised residues 212–579, and the second included residues 241–579, with an internal deletion of residues 275–357. All constructs were subcloned into a modified pET28a vector (Novagen) (GenBank accessions EF442785 and EF456735).

GAL4 fusion constructs for REV-ERBα and REV-ERBβ were generated by first cloning the Gal4 DNA-binding domain (DBD) (amino acids 1–132) into pBluescript II (Stratagene), containing an SV40 3′UTR. PmeI and NheI restriction sites were introduced as cloning sites for NR LBD introduction. The following primers were used to amplify and clone the Rev-erbα LBD (aa 215–610; 5′-ATTAGCTAGCATGCTTGCTGAGATGCAGAGTGCC and 3′-ATTAGTTTAAACCTACTAGTCCACCCGGAAGGACAGCAGC), Rev-erbβ (aa 223–577; 5′-ATTAGCTAGCGCCCAGGAACAGCTGCGACCCAAGCC and 3′-ATTAGTTTAAACCTACTAAACTTTAAAGGCCAAGAGCTCC) into pBS Gal1 NheI-PmeI. The Gal4-NR cDNA was then subcloned into pcDNA 3.1 V5/His using HindIII-PmeI restriction sites.

### Protein purification and crystallization.

Hexahistidine-tagged proteins were expressed in *E. Coli* (BL21-Gold[DE3] pLysS; Stratagene) grown in 1 l of either Terrific Broth or selenomethionine medium [92] in the presence of 50 μg/ml kanamycin, 25 μg/ml chloramphenicol, and in the absence or presence of hemin (12.5 μM; Sigma. Cells were grown at 37 °C to an OD_600_ of 1.2, and, following the addition of isopropyl-1-thio-D-galactopyranoside (final concentration 1 mM) to induce expression, the cells were incubated overnight with shaking at 25 °C. Following centrifugation, the cell paste was resuspended in 30 ml binding buffer (5 mM imidazole, 500 mM NaCl, 0.5 mM TCEP, 5% glycerol 50 mM Hepes [pH 7.5]) and sonicated on ice (3-s intervals) for 5 min. Protein was purified from clarified supernatant using Ni-NTA affinity chromatography (*column volume* 3 ml). Once loaded, the column was washed with 300 ml of buffer containing 30 mM imidazole, 500 mM NaCl, 5% glycerol, 0.5 mM TCEP (*tris*(2-carboxyethyl)phosphine), and 50 mM Hepes (pH 7.5). Protein was eluted from the column using an equivalent buffer containing 250 mM imidazole, and dialysed overnight into a buffer containing 500 mM NaCl, 0.5 mM TCEP, and 50 mM Hepes (pH 7.5). Using hemin supplemented protein, in situ proteolysis of REV-ERBβ (241–579 Δ 275–357, 17 mg/ml or 212–579, 13.8 mg/ml) was crystallized using the hanging drop vapor diffusion method at 22 °C by mixing 2 μl of the protein solution with 2 μl of the reservoir solution containing 1.6 M ammonium sulfate, 0.1 M Na Hepes (pH 7.6), 4% Jeffamine M-600, and was performed in the crystallization drop by adding a 1:2,000 ratio (w/w) of trypsin (1.5 mg/ml; Sigma) to the protein [47].

### Spectroscopic analysis.

Heme-bound REV-ERBβ (17 μM) was reduced to the Fe(II) state using 2 mM dithionite. A CO stock solution was prepared by saturating degassed storage buffer (10 mM Tris [pH 8.0] at 4 °C, 500 mM NaCl) with CO (Praxair). Similarly, NO stock solution was prepared using degassed storage buffer supplemented with 200 mM Tris-HCl saturated with NO gas (Aldrich), then adjusted to (pH 8.0). The estimated concentration of CO and NO in the stock solutions was 1 mM and 1.9 mM, respectively, based on the mole fraction solubility of the gases in water [93]. The gases were then added to the protein samples at a final concentration of 100 μM, providing an approximate 5-fold molar excess of gas in solution.

### Removal of heme from receptors.

To test dissociation of heme, heme-bound forms of both REV-ERB LBDs (4 mg) were bound to Ni-NTA agarose beads (Qiagen) through hexahistidine tags. Proteins were washed for 12 h at a rate of 1 ml/min using a buffer containing 150 mM NaCl, 10 mM Tris (pH 8.3), 5% glycerol, and 0.1% Triton X-100. Proteins were eluted from Ni beads using the same buffer supplemented with 500 mM imidazole. Electronic absorption spectra were taken of washed and unwashed reference samples at equivalent protein concentration to compare heme content.

### X-ray crystallographic analysis.

Data from flash-cooled crystals of the Se-Met REV-ERBβ/heme complex were collected at 0.97 Å at 100 K at the APS at Argonne National Laboratory (SER CAT, beamline 19ID), and the data integrated and scaled to 1.9 Å resolution by using DENZO/SCALEPACK [94]. The structure was solved by molecular replacement using the apo REV-ERBβ structure (PDB IDs: 2V7C, 2V0V) [21] as a search model using PHASER [95]. Structures were initially traced by ARP/WARP [96] and then manually rebuilt in COOT [97]. Final refinement was performed by using REFMAC [98]. Additional crystallographic statistics are given in Table S2. The drawings were generated with PYMOL [99]. Solvent exposure of heme in the REV-ERBβ structure was calculated using Swiss PDB viewer [100]. Heme volume calculations were made using the Java molecular editor (Peter Ertl, Novartis) using the smiles descriptors: Heme, CC1 = C(C2 = CC3 = C(C(= C([N]3)C = C4C(= C(C(= N4) C = C5C(= C(C(= N5)C = C1[N]2) C = C)C)C = C) C)C)CCC(= O)O)CCC(= O)O.[Fe+3] (NCBI Pubchem). Modeling of the SMRT co-repressor motif with both REV-ERB structures was done using PYMOL. REV-ERB/SMRT models were generated by structural alignment of the REV-ERB structures (PDB IDs: 2V0V, [21]; 3CQV) with the structure of antagonist-bound PPARα in complex with SMRT peptide (PDB: 1KKQ, [101]). The structure of the PPARα LBD and antagonist were then removed, leaving the model of each REV-ERB structure with the SMRT co-repressor motif.

### Cell culture and treatments.

HEK 293T and HepG2 cells were grown in Dulbecco's modified Eagle's medium (Wisent) supplemented with 10% fetal bovine serum (Sigma), 2 mM L-glutamine, 100 IU/ml penicillin, 0.1 mg/ml streptomycin, and nonessential amino acids (Invitrogen). Cells were incubated at 37 °C in 5% CO_2_ and, upon harvest, washed with PBS (Wisent). Gas treatment of cells was performed in the presence of 25 μM Hemin (Sigma) and used the NO donors Deta/NO (300 μM, Sigma) freshly dissolved in 10 mM NaOH, or two doses of SNAP (200 μM, Sigma), dissolved in 5 mM EDTA and 10 mM PBS, separated by 5 h. CO treatment of cells was done by culturing cells in sealed chambers with 5% CO_2_ and 0.05% (500 ppm) − 0.2% (2,000 ppm) CO (Praxair) at 37 °C. Prior to transfer of cells to chambers, culture medium was spiked with a volume of CO-saturated buffer equivalent to their respective treatment concentration. All experiments were conducted a minimum of three times in triplicate, and the mean +/− standard deviation of a representative experiment is shown.

### Luciferase-LBD reporter assays.

Using antibiotic free medium, 293T cells were seeded at a density of 2 × 10^5^ cells per well in 24-well plates 1 d prior to transfection. Transfection was carried out using Lipofectamine 2000 (Invitrogen) as described in the manufacture's instructions. Unless otherwise stated, transfections contained 400 ng of 2X-UAS luciferase reporter, 50 ng pSV40 β-gal, 50 ng of either Rev-erbα-Gal4 or Rev-erbβ-Gal4 and pSP empty vector to a total of 800 ng DNA per well. REV-ERBα-GAL4 and REV-ERBβ-GAL4 were expressed using the modified pcDNA 3.1 plasmid described above; NCOR, luciferase, and β-GAL constructs were expressed as previously described [102,103]; and the pI RIP140 was a kind gift from M.G. Parker. Compound treatments were applied 6 h post-transfection as described above and cells harvested 24 h later. Luciferase values were normalized to β-GAL activities as described previously and are presented as a ratio of the reporter alone under equivalent conditions [19]. In the case of Deta/NO treatments, luciferase values were also normalized to GAL4 only reporter controls.

### RNA interference assay.

HEK 293T cells were transfected with siRNA using Lipofectamine 2000 (Invitrogen) according to the manufacturer's protocol. For each targeted gene, a pool of four siRNAs (Dharmacon) with the following sequences were used. Rev-erbα: GCAUGGACGCAGUGGGCGAUU, GGGCAUGUCUCGAGACGCUUU, CGGCAGGGCAACUCAAAGAUU, GGGCGAACGGUGCAGGAGAUU; Rev-erbβ: GAAGAAUGAUC GAAUAGAUUU, GAACAUGGAGCAAUAUAAUUU, GAGGAGCUCUUGGCCUUUAUU, and UAAAC AACAUGCACUCUGAUU. Experimental treatments were completed between 24 h and 96 h of siRNA transfection.

### Quantitative Real Time-PCR.

Total RNA was isolated from 1 to 2.5 × 10^6^ cells using the RNeasy mini kit (Qiagen) according to manufacturer's protocol. 2 μg RNA samples were individually treated with DNase I (Fermentas) and then reverse-transcribed to synthesize cDNA using pd(N)6-random hexamer primers (Promega) and RevertAid H Minus M-MuLV Reverse Transcriptase (Fermentas). Quantitative PCR was performed in triplicate for each sample with SYBR Green (Sigma) using the ABI Prism 7000 sequence detection system (Applied Biosystems). Transcript levels were determined using the comparative C_t_ method with β-actin as reference. Primer sequences used were as follows: *Rev-erb*α, forward, ACTTCCCACCATCCCCCACT, reverse, GGAAGAAGGGGAGCCGTCAT [15]; *Rev-erb*β, forward, TCTTGTCACAGTGAGGGTTCT, reverse, GCGAGATCACCATTCTTGGGA; *Bmal1*, forward, GAAAAGCGGCGTCGGGATAA, reverse, GGACATTGCGTTGCATGTTGG [104]; and β*-actin*, forward, TGGACTTCGAGCAAGAGATGG, reverse, GGAAGGAAGGCTGGAAGAGTG [105].

### Fluorescence polarization.

Using the purified REV-ERB LDB constructs, peptide interaction was monitored using fluorescence polarization. N-terminally fluorescein-labeled peptides, corresponding to interaction domain I for NCOR (110 nM; DPASNLGLEDIIRKALMGSF) and SMRT (110 nM; ASTNMGLEAIIRKALMGKYD), were combined with a dilution series of either LBD in a buffer of 100 mM Tris (pH 8.2) and 150 mM NaCl. Bacterial expression of the LBDs was done in the presence or absence of supplemental heme, as described above, resulting in either apo- or holo-forms of the receptors. Gas treatment of solutions was done using the NO donor SNAP (1,200 μM). Detection of changes in polarization was measured using a Biotek Synergy 2 plate reader (λ_excitation_ = 485 nm, λ_emission_ = 528 nm) in 384-well format using black PCR microplates (Axygen). Fluorescence polarization, in millipolarization (mP) units, was calculated as mP = (I − I_⊥_) / (I + I_⊥_) × 1,000 where I = parallel emission intensity measurement and I_⊥_ = perpendicular emission intensity measurement. Increases in mP units reflect increased binding of the peptide to the REV-ERB LBDs. Measurements in graphs are in triplicate and represent a single time point during a 5-h incubation. Binding parameters were calculated by nonlinear curve fitting using the one site binding (hyperbola) formula *Y* = *Bmax* × *X*/(*Kd* + *X*) (GraphPad Prism version 4.0 for window, GraphPad Software).

## Supporting Information

Figure S1 Bacterial Expressed and Purified REV-ERBα and β LBDs Increased supplementation of bacterial cultures with hemin leads to greater heme occupancy with no effect on stability. (A) REV-ERBα and (B) REV-ERBβ LBDs after recombinant expression and purification with Ni affinity resin. Lane 1, no supplementation; lane 2, [hemin] = 0.05 μM; lane 3, [hemin] = 0.20 μM; lane 4, [hemin] = 0.40 μM.
(3.43 MB TIF)

Figure S2 Washing of Heme-Bound REV-ERBα (A) and β (B) LBDs Leads to Depletion of Heme Occupancy At equal protein concentration (280 nm), electronic absorption spectra for washed REV-ERB LBDs show a reduction in the characteristic hemoprotein γ peak (413 nm).
(293 KB TIF)

Figure S3 Quantitative Real-Time PCR Analysis of Bmal1 and Rev-erbα and β Transcript Levels in HepG2 Cells Endogenous mRNA levels of Bmal1 and Rev-erbα and β under control and 300 μM Deta/NO treatments.
(53 KB TIF)

Figure S4 Sequence Alignment of Human REV-ERBα, Human REV-ERBβ, and REV-ERBβ Homologues in Other Metazoans Sequences were aligned using Clustal W [106] and then adjusted manually. Determined secondary structure of heme-bound REV-ERBβ is labeled above and residues involved in heme coordination are marked (*). All residues that could coordinate heme in the loop and His, Cys through the proteins are in bold type. Dashed line, Cys/Pro motifs of putative heme responsive motifs; solid line, HXXC motifs.
(177 KB TIF)

Table S1 Residues within 4 Å of Heme in REV-ERBβ Structure Residues have been categorized based on whether they interaction with the heme face or heme edge. The table lists position, distance from heme, chemical property, and hydropathy index for each residue [107].
(487 KB TIF)

Table S2 Data Collection and Solution Structure Parameters (38 KB DOC)

## Abbreviations

BMAL1: Brain, Muscle Arnt-like protein-1
CO: carbon monoxide
Deta/NO: diethylenetriamine/NO
E75: Ecdysone-induced protein 75
HEK: human embryonic kidney
HepG2: hepatocellular carcinoma
LBD: ligand-binding domain
mPER2: mammalian Period 2
mP: millipolarization unit
NCOR: Nuclear Receptor Co-repressor
NO: nitric oxide
NPAS2: Neuronal PAS domain protein
NR: nuclear receptor
RIP140: Receptor Interaction Protein 140
ROR: Retinoid-related Orphan Receptor
ROS: reactive oxidant species
siRNA: small interfering RNA
SMRT: Silencing Mediator for Retinoid and Thyroid hormone receptor
SNAP: S-nitroso-N-acetyl-l,l-penicillamine
TCEP: tris(2-carboxyethyl)phosphine
UAS: upstream activating sequences

## References

[pbio-1000043-b001] Harding HP, Lazar MA (1995). The monomer-binding orphan receptor Rev-Erb represses transcription as a dimer on a novel direct repeat. Mol Cell Biol.

[pbio-1000043-b002] Giguere V (1999). Orphan nuclear receptors: from gene to function. Endocr Rev.

[pbio-1000043-b003] Benoit G, Cooney A, Giguere V, Ingraham H, Lazar M (2006). International Union of Pharmacology. LXVI. Orphan nuclear receptors. Pharmacol Rev.

[pbio-1000043-b004] Forman BM, Chen J, Blumberg B, Kliewer SA, Henshaw R (1994). Cross-talk among ROR alpha 1 and the Rev-erb family of orphan nuclear receptors. Mol Endocrinol.

[pbio-1000043-b005] Giguere V, Tini M, Flock G, Ong E, Evans RM (1994). Isoform-specific amino-terminal domains dictate DNA-binding properties of ROR alpha, a novel family of orphan hormone nuclear receptors. Genes Dev.

[pbio-1000043-b006] Guillaumond F, Dardente H, Giguere V, Cermakian N (2005). Differential control of Bmal1 circadian transcription by REV-ERB and ROR nuclear receptors. J Biol Rhythms.

[pbio-1000043-b007] Storch KF, Lipan O, Leykin I, Viswanathan N, Davis FC (2002). Extensive and divergent circadian gene expression in liver and heart. Nature.

[pbio-1000043-b008] Panda S, Antoch MP, Miller BH, Su AI, Schook AB (2002). Coordinated transcription of key pathways in the mouse by the circadian clock. Cell.

[pbio-1000043-b009] Balsalobre A, Damiola F, Schibler U (1998). A serum shock induces circadian gene expression in mammalian tissue culture cells. Cell.

[pbio-1000043-b010] Torra IP, Tsibulsky V, Delaunay F, Saladin R, Laudet V (2000). Circadian and glucocorticoid regulation of Rev-erbalpha expression in liver. Endocrinology.

[pbio-1000043-b011] Preitner N, Damiola F, Lopez-Molina L, Zakany J, Duboule D (2002). The orphan nuclear receptor REV-ERBalpha controls circadian transcription within the positive limb of the mammalian circadian oscillator. Cell.

[pbio-1000043-b012] Vu-Dac N, Chopin-Delannoy S, Gervois P, Bonnelye E, Martin G (1998). The nuclear receptors peroxisome proliferator-activated receptor alpha and Rev-erbalpha mediate the species-specific regulation of apolipoprotein A-I expression by fibrates. J Biol Chem.

[pbio-1000043-b013] Coste H, Rodriguez JC (2002). Orphan nuclear hormone receptor Rev-erbalpha regulates the human apolipoprotein CIII promoter. J Biol Chem.

[pbio-1000043-b014] Raspe E, Duez H, Mansen A, Fontaine C, Fievet C (2002). Identification of Rev-erbalpha as a physiological repressor of apoC-III gene transcription. J Lipid Res.

[pbio-1000043-b015] Migita H, Morser J, Kawai K (2004). Rev-erbalpha upregulates NF-kappaB-responsive genes in vascular smooth muscle cells. FEBS Letters.

[pbio-1000043-b016] Delerive P, Monte D, Dubois G, Trottein F, Fruchart-Najib J (2001). The orphan nuclear receptor ROR alpha is a negative regulator of the inflammatory response. EMBO Rep.

[pbio-1000043-b017] Yin L, Wu N, Curtin JC, Qatanani M, Szwergold NR (2007). Rev-erb{alpha}, a heme sensor that coordinates metabolic and circadian pathways. Science.

[pbio-1000043-b018] White KP, Hurban P, Watanabe T, Hogness DS (1997). Coordination of Drosophila metamorphosis by two ecdysone-induced nuclear receptors. Science.

[pbio-1000043-b019] Reinking J, Lam MM, Pardee K, Sampson HM, Liu S (2005). The Drosophila nuclear receptor e75 contains heme and is gas responsive.[see comment]. Cell.

[pbio-1000043-b020] Raghuram S, Stayrook KR, Huang P, Rogers PM, Nosie AK (2007). Identification of heme as the ligand for the orphan nuclear receptors REV-ERBalpha and REV-ERBbeta. Nat Struct Mol Biol.

[pbio-1000043-b021] Woo EJ, Jeong DG, Lim MY, Jun Kim S, Kim KJ (2007). Structural insight into the constitutive repression function of the nuclear receptor Rev-erbbeta. J Mol Biol.

[pbio-1000043-b022] Ding JM, Chen D, Weber ET, Faiman LE, Rea MA (1994). Resetting the biological clock: mediation of nocturnal circadian shifts by glutamate and NO. Science.

[pbio-1000043-b023] Dioum EM, Rutter J, Tuckerman JR, Gonzalez G, Gilles-Gonzalez MA (2002). NPAS2: a gas-responsive transcription factor. Science.

[pbio-1000043-b024] DeBruyne JP, Weaver DR, Reppert SM (2007). CLOCK and NPAS2 have overlapping roles in the suprachiasmatic circadian clock. Nat Neurosci.

[pbio-1000043-b025] Hogenesch JB, Gu YZ, Jain S, Bradfield CA (1998). The basic-helix-loop-helix-PAS orphan MOP3 forms transcriptionally active complexes with circadian and hypoxia factors. Proc Natl Acad Sci U S A.

[pbio-1000043-b026] Gekakis N, Staknis D, Nguyen HB, Davis FC, Wilsbacher LD (1998). Role of the CLOCK protein in the mammalian circadian mechanism. Science.

[pbio-1000043-b027] Kume K, Zylka MJ, Sriram S, Shearman LP, Weaver DR (1999). mCRY1 and mCRY2 are essential components of the negative limb of the circadian clock feedback loop. Cell.

[pbio-1000043-b028] Reick M, Garcia JA, Dudley C, McKnight SL (2001). NPAS2: an analog of clock operative in the mammalian forebrain. Science.

[pbio-1000043-b029] Triqueneaux G, Thenot S, Kakizawa T, Antoch MP, Safi R (2004). The orphan receptor Rev-erbalpha gene is a target of the circadian clock pacemaker. J Mol Endocrinol.

[pbio-1000043-b030] de Rosny E, de Groot A, Jullian-Binard C, Gaillard J, Borel F (2006). Drosophila nuclear receptor E75 is a thiolate hemoprotein. Biochemistry.

[pbio-1000043-b031] Shelver D, Kerby RL, He Y, Roberts GP (1997). CooA, a CO-sensing transcription factor from Rhodospirillum rubrum, is a CO-binding heme protein. Proc Natl Acad Sci U S A.

[pbio-1000043-b032] Zheng M, Storz G (2000). Redox sensing by prokaryotic transcription factors. Biochem Pharmacol.

[pbio-1000043-b033] Uchida T, Sato E, Sato A, Sagami I, Shimizu T (2005). CO-dependent activity-controlling mechanism of heme-containing CO-sensor protein, neuronal PAS domain protein 2. J Biol Chem.

[pbio-1000043-b034] Marvin K, Reinking JL, Lee AJ, Pardee K, Krause HM (2008). Nuclear receptors Homo sapiens Rev-erbβ and Drosophila melanogaster E75 are thiolate-ligated heme proteins, which undergo redox-mediated ligand switching and bind CO and NO. Biochemistry.

[pbio-1000043-b035] Shelver D, Thorsteinsson MV, Kerby RL, Chung SY, Roberts GP (1999). Identification of two important heme site residues (cysteine 75 and histidine 77) in CooA, the CO-sensing transcription factor of Rhodospirillum rubrum. Biochemistry.

[pbio-1000043-b036] Inagaki S, Masuda C, Akaishi T, Nakajima H, Yoshioka S (2005). Spectroscopic and redox properties of a CooA homologue from Carboxydothermus hydrogenoformans. J Biol Chem.

[pbio-1000043-b037] Roberts GP, Kerby RL, Youn H, Conrad M (2005). CooA, a paradigm for gas sensing regulatory proteins. J Inorg Biochem.

[pbio-1000043-b038] Marvin KA, Kerby RL, Youn H, Roberts GP, Burstyn JN (2008). The transcription regulator RcoM-2 from Burkholderia xenovorans is a cysteine-ligated hemoprotein that undergoes a redox-mediated ligand switch. Biochemistry.

[pbio-1000043-b039] Stone JR, Marletta MA (1994). Soluble guanylate cyclase from bovine lung: activation with nitric oxide and carbon monoxide and spectral characterization of the ferrous and ferric states. Biochemistry.

[pbio-1000043-b040] Reynolds MF, Parks RB, Burstyn JN, Shelver D, Thorsteinsson MV (2000). Electronic absorption, EPR, and resonance raman spectroscopy of CooA, a CO-sensing transcription activator from R. rubrum, reveals a five-coordinate NO-heme. Biochemistry.

[pbio-1000043-b041] Yin L, Wang J, Klein PS, Lazar MA (2006). Nuclear receptor Rev-erbalpha is a critical lithium-sensitive component of the circadian clock. Science.

[pbio-1000043-b042] Kaasik K, Lee CC (2004). Reciprocal regulation of haem biosynthesis and the circadian clock in mammals. Nature.

[pbio-1000043-b043] Rogers PM, Ying L, Burris TP (2008). Relationship between circadian oscillations of Rev-erbalpha expression and intracellular levels of its ligand, heme. Biochem Biophys Res Commun.

[pbio-1000043-b044] Ishizuka T, Lazar MA (2003). The N-CoR/histone deacetylase 3 complex is required for repression by thyroid hormone receptor. Mol Cell Biol.

[pbio-1000043-b045] Farooq M, Sulochana KN, Pan X, To J, Sheng D (2008). Histone deacetylase 3 (hdac3) is specifically required for liver development in zebrafish. Dev Biol.

[pbio-1000043-b046] Wei LN, Hu X, Chandra D, Seto E, Farooqui M (2000). Receptor-interacting protein 140 directly recruits histone deacetylases for gene silencing. J Biol Chem.

[pbio-1000043-b047] Dong A, Xu X, Edwards AM, Chang C, Chruszcz M (2007). In situ proteolysis for protein crystallization and structure determination. Nat Methods.

[pbio-1000043-b048] Schneider S, Marles-Wright J, Sharp KH, Paoli M (2007). Diversity and conservation of interactions for binding heme in b-type heme proteins. Nat Prod Rep.

[pbio-1000043-b049] Weatherman RV, Fletterick RJ, Scanlan TS (1999). Nuclear-receptor ligands and ligand-binding domains. Annu Rev Biochem.

[pbio-1000043-b050] Wang J, Luthey-Schulten ZA, Suslick KS (2003). Is the olfactory receptor a metalloprotein?. Proc Natl Acad Sci U S A.

[pbio-1000043-b051] Lathrop JT, Timko MP (1993). Regulation by heme of mitochondrial protein transport through a conserved amino acid motif. Science.

[pbio-1000043-b052] Zhang L, Guarente L (1995). Heme binds to a short sequence that serves a regulatory function in diverse proteins. Embo J.

[pbio-1000043-b053] Lee HC, Hon T, Lan C, Zhang L (2003). Structural environment dictates the biological significance of heme-responsive motifs and the role of Hsp90 in the activation of the heme activator protein Hap1. Mol Cell Biol.

[pbio-1000043-b054] Schalk M, Nedelkina S, Schoch G, Batard Y, Werck-Reichhart D (1999). Role of unusual amino acid residues in the proximal and distal heme regions of a plant P450, CYP73A1. Biochemistry.

[pbio-1000043-b055] Huang TJ, McCoubrey WK, Maines MD (2001). Heme oxygenase-2 interaction with metalloporphyrins: function of heme regulatory motifs. Antioxid Redox Signal.

[pbio-1000043-b056] Igarashi J, Murase M, Iizuka A, Pichierri F, Martinkova M (2008). Elucidation of the heme binding site of heme-regulated eukaryotic initiation factor 2alpha kinase and the role of the regulatory motif in heme sensing by spectroscopic and catalytic studies of mutant proteins. J Biol Chem.

[pbio-1000043-b057] Burke L, Downes M, Carozzi A, Giguere V, Muscat GE (1996). Transcriptional repression by the orphan steroid receptor RVR/Rev-erb beta is dependent on the signature motif and helix 5 in the E region: functional evidence for a biological role of RVR in myogenesis. Nucleic Acids Res.

[pbio-1000043-b058] Downes M, Burke LJ, Muscat GE (1996). Transcriptional repression by Rev-erbA alpha is dependent on the signature motif and helix 5 in the ligand binding domain: silencing does not involve an interaction with N-CoR. Nucleic Acids Res.

[pbio-1000043-b059] Burke LJ, Downes M, Laudet V, Muscat GE (1998). Identification and characterization of a novel corepressor interaction region in RVR and Rev-erbA alpha. Mol Endocrinol.

[pbio-1000043-b060] Renaud JP, Harris JM, Downes M, Burke LJ, Muscat GE (2000). Structure-function analysis of the Rev-erbA and RVR ligand-binding domains reveals a large hydrophobic surface that mediates corepressor binding and a ligand cavity occupied by side chains. Mol Endocrinol.

[pbio-1000043-b061] Billas IM, Iwema T, Garnier JM, Mitschler A, Rochel N (2003). Structural adaptability in the ligand-binding pocket of the ecdysone hormone receptor. Nature.

[pbio-1000043-b062] Mitro N, Mak PA, Vargas L, Godio C, Hampton E (2007). The nuclear receptor LXR is a glucose sensor. Nature.

[pbio-1000043-b063] Nettles KW, Bruning JB, Gil G, O'Neil EE, Nowak J (2007). Structural plasticity in the oestrogen receptor ligand-binding domain. EMBO Rep.

[pbio-1000043-b064] Mannick JB, Schonhoff CM (2002). Nitrosylation: the next phosphorylation?. Arch Biochem Biophys.

[pbio-1000043-b065] Zamir I, Harding HP, Atkins GB, Horlein A, Glass CK (1996). A nuclear hormone receptor corepressor mediates transcriptional silencing by receptors with distinct repression domains. Mol Cell Biol.

[pbio-1000043-b066] White R, Morganstein D, Christian M, Seth A, Herzog B (2007). Role of RIP140 in metabolic tissues: connections to disease. FEBS Lett.

[pbio-1000043-b067] Herzog B, Hallberg M, Seth A, Woods A, White R (2007). The nuclear receptor cofactor, receptor-interacting protein 140, is required for the regulation of hepatic lipid and glucose metabolism by liver X receptor. Mol Endocrinol.

[pbio-1000043-b068] Kerley JS, Olsen SL, Freemantle SJ, Spinella MJ (2001). Transcriptional activation of the nuclear receptor corepressor RIP140 by retinoic acid: a potential negative-feedback regulatory mechanism. Biochem Biophys Res Commun.

[pbio-1000043-b069] Chaturvedi P, Pratta M, Steplewski K, Connor J, Kumar S (2006). Functional characterization of an orphan nuclear receptor, Rev-ErbAalpha, in chondrocytes and its potential role in osteoarthritis. Arthritis Rheum.

[pbio-1000043-b070] Ramakrishnan SN, Lau P, Burke LJ, Muscat GE (2005). Rev-erbbeta regulates the expression of genes involved in lipid absorption in skeletal muscle cells: evidence for cross-talk between orphan nuclear receptors and myokines. J Biol Chem.

[pbio-1000043-b071] Seth A, Steel JH, Nichol D, Pocock V, Kumaran MK (2007). The transcriptional corepressor RIP140 regulates oxidative metabolism in skeletal muscle. Cell Metab.

[pbio-1000043-b072] Schibler U, Naef F (2005). Cellular oscillators: rhythmic gene expression and metabolism. Curr Opin Cell Biol.

[pbio-1000043-b073] Laitinen S, Fontaine C, Fruchart JC, Staels B (2005). The role of the orphan nuclear receptor Rev-Erb alpha in adipocyte differentiation and function. Biochimie.

[pbio-1000043-b074] Ramakrishnan SN, Muscat GEO (2006). The orphan Rev-erb nuclear receptors: a link between metabolism, circadian rhythm and inflammation?. Nucl Recept Signal.

[pbio-1000043-b075] Paoli M, Marles-Wright J, Smith A (2002). Structure-function relationships in heme-proteins. DNA Cell Biol.

[pbio-1000043-b076] Yang J, Kim KD, Lucas A, Drahos KE, Santos CS (2008). A novel heme-regulatory motif mediates heme-dependent degradation of the circadian factor period 2. Mol Cell Biol.

[pbio-1000043-b077] Tsiftsoglou AS, Tsamadou AI, Papadopoulou LC (2006). Heme as key regulator of major mammalian cellular functions: molecular, cellular, and pharmacological aspects. Pharmacol Ther.

[pbio-1000043-b078] Pardee K, Reinking J, Krause H (2004). Nuclear hormone receptors, metabolism, and aging: what goes around comes around.Transcription factors link lipid metabolism and aging-related processes. Sci Aging Knowledge Environ.

[pbio-1000043-b079] Zheng B, Albrecht U, Kaasik K, Sage M, Lu W (2001). Nonredundant roles of the mPer1 and mPer2 genes in the mammalian circadian clock. Cell.

[pbio-1000043-b080] Rubio MF, Agostino PV, Ferreyra GA, Golombek DA (2003). Circadian heme oxygenase activity in the hamster suprachiasmatic nuclei. Neurosci Lett.

[pbio-1000043-b081] Ferreyra GA, Cammarota MP, Golombek DA (1998). Photic control of nitric oxide synthase activity in the hamster suprachiasmatic nuclei. Brain Res.

[pbio-1000043-b082] Nathan C (1992). Nitric oxide as a secretory product of mammalian cells. FASEB J.

[pbio-1000043-b083] Aberg AM, Hultin M, Abrahamsson P, Larsson JE (2004). Circulatory effects and kinetics following acute administration of carbon monoxide in a porcine model. Life Sci.

[pbio-1000043-b084] Hardeland R, Coto-Montes A, Poeggeler B (2003). Circadian rhythms, oxidative stress, and antioxidative defense mechanisms. Chronobiol Int.

[pbio-1000043-b085] Tu BP, McKnight SL (2006). Metabolic cycles as an underlying basis of biological oscillations. Nat Rev Mol Cell Biol.

[pbio-1000043-b086] Winterbourn CC (2008). Reconciling the chemistry and biology of reactive oxygen species. Nat Chem Biol.

[pbio-1000043-b087] Winterbourn CC, Hampton MB (2008). Thiol chemistry and specificity in redox signaling. Free Radic Biol Med.

[pbio-1000043-b088] Walker E, Mittal V, Tessner K (2008). Stress and the hypothalamic pituitary adrenal axis in the developmental course of schizophrenia. Annu Rev Clin Psychol.

[pbio-1000043-b089] Bao AM, Meynen G, Swaab DF (2008). The stress system in depression and neurodegeneration: focus on the human hypothalamus. Brain Res Rev.

[pbio-1000043-b090] Rutter J, Reick M, Wu LC, McKnight SL (2001). Regulation of clock and NPAS2 DNA binding by the redox state of NAD cofactors. Science.

[pbio-1000043-b091] Dong G, Golden SS (2008). How a cyanobacterium tells time. Curr Opin Microbiol.

[pbio-1000043-b092] Porciero S, Receveur-Brechot V, Mori K, Franzetti B, Roussel A (2005). Expression, purification, crystallization and preliminary crystallographic analysis of a deblocking aminopeptidase from Pyrococcus horikoshii. Acta Crystallogr Sect F Struct Biol Cryst Commun.

[pbio-1000043-b093] Gevantman L, Lide DR (2004). Solubility of selected gases in water. CRC handbook of chemistry and physics.

[pbio-1000043-b094] Otwinowski ZM, Minor W, Carter CW, Sweet RM (1997). Processing of X-ray diffraction data collected in oscillation mode. Methods in enzymology, volume 276: macromolecular crystallography, part A.

[pbio-1000043-b095] McCoy AJ (2007). Solving structures of protein complexes by molecular replacement with Phaser. Acta Crystallogr D Biol Crystallogr.

[pbio-1000043-b096] Perrakis A, Harkiolaki M, Wilson KS, Lamzin VS (2001). ARP/wARP and molecular replacement. Acta Crystallogr D Biol Crystallogr.

[pbio-1000043-b097] Emsley P, Cowtan K (2004). Coot: model-building tools for molecular graphics. Acta Crystallogr D Biol Crystallogr.

[pbio-1000043-b098] Murshudov GN, Vagin AA, Dodson EJ (1997). Refinement of macromolecular structures by the maximum-likelihood method. Acta Crystallogr D Biol Crystallogr.

[pbio-1000043-b099] DeLano W (2002). The PyMOL Molecular Graphics System.

[pbio-1000043-b100] Guex N, Peitsch MC (1997). SWISS-MODEL and the Swiss-PdbViewer: an environment for comparative protein modeling. Electrophoresis.

[pbio-1000043-b101] Xu HE, Stanley TB, Montana VG, Lambert MH, Shearer BG (2002). Structural basis for antagonist-mediated recruitment of nuclear co-repressors by PPARalpha. Nature.

[pbio-1000043-b102] Tiefenbach J, Novac N, Ducasse M, Eck M, Melchior F (2006). SUMOylation of the corepressor N-CoR modulates its capacity to repress transcription. Mol Biol Cell.

[pbio-1000043-b103] Mangelsdorf DJ, Umesono K, Kliewer SA, Borgmeyer U, Ong ES (1991). A direct repeat in the cellular retinol-binding protein type II gene confers differential regulation by RXR and RAR. Cell.

[pbio-1000043-b104] Wang X, Seed B (2003). A PCR primer bank for quantitative gene expression analysis. Nucleic Acids Res.

[pbio-1000043-b105] Randall G, Chen L, Panis M, Fischer AK, Lindenbach BD (2006). Silencing of USP18 potentiates the antiviral activity of interferon against hepatitis C virus infection. Gastroenterology.

[pbio-1000043-b106] Chenna R, Sugawara H, Koike T, Lopez R, Gibson TJ (2003). Multiple sequence alignment with the Clustal series of programs. Nucleic Acids Res.

[pbio-1000043-b107] Kyte J, Doolittle RF (1982). A simple method for displaying the hydropathic character of a protein. J Mol Biol.

