# Supplementary figures and images for: The Structural Basis of Gas-Responsive Transcription by the Human Nuclear Hormone Receptor REV-ERBβ
Source: PLoS Biol. 2009 Feb 24;7(2):e1000043. doi: 10.1371/journal.pbio.1000043 (PMC2652392; doi:10.1371/journal.pbio.1000043)

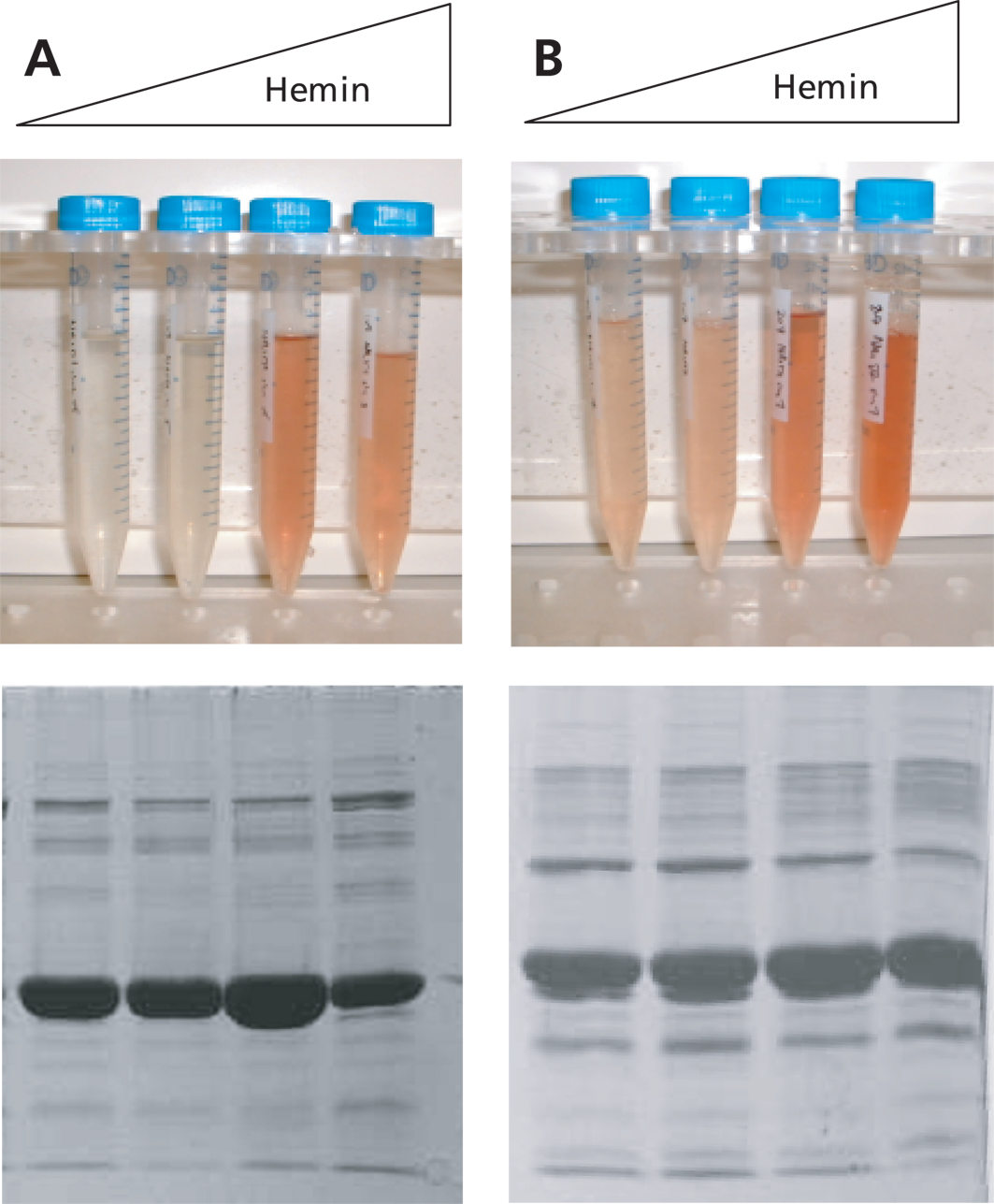

Supplement: Figure S1 — Increased supplementation of bacterial cultures with hemin leads to greater heme occupancy with no effect on stability. (A) REV-ERBα and (B) REV-ERBβ LBDs after recombinant expression and purification with Ni affinity resin. Lane 1, no supplementation; lane 2, [hemin] = 0.05 μM; lane 3, [hemin] = 0.20 μM; lane 4, [hemin] = 0.40 μM. (3.43 MB TIF) [file pbio.1000043.sg001.tif]

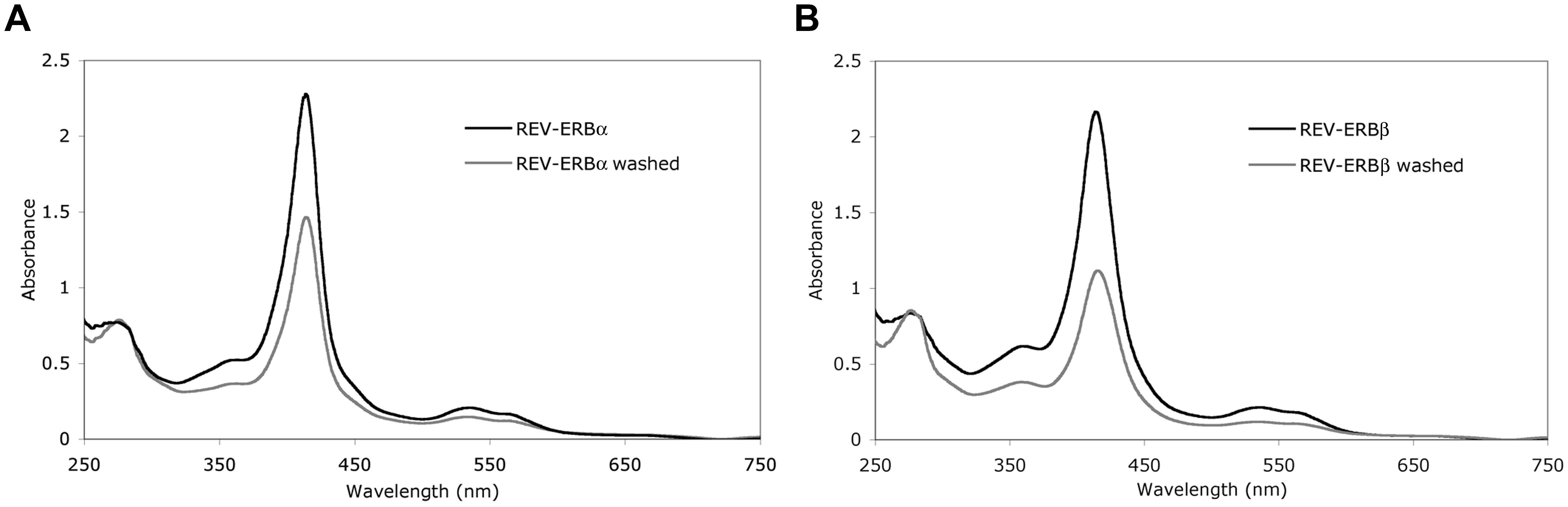

Supplement: Figure S2 — At equal protein concentration (280 nm), electronic absorption spectra for washed REV-ERB LBDs show a reduction in the characteristic hemoprotein γ peak (413 nm). (293 KB TIF) [file pbio.1000043.sg002.tif]

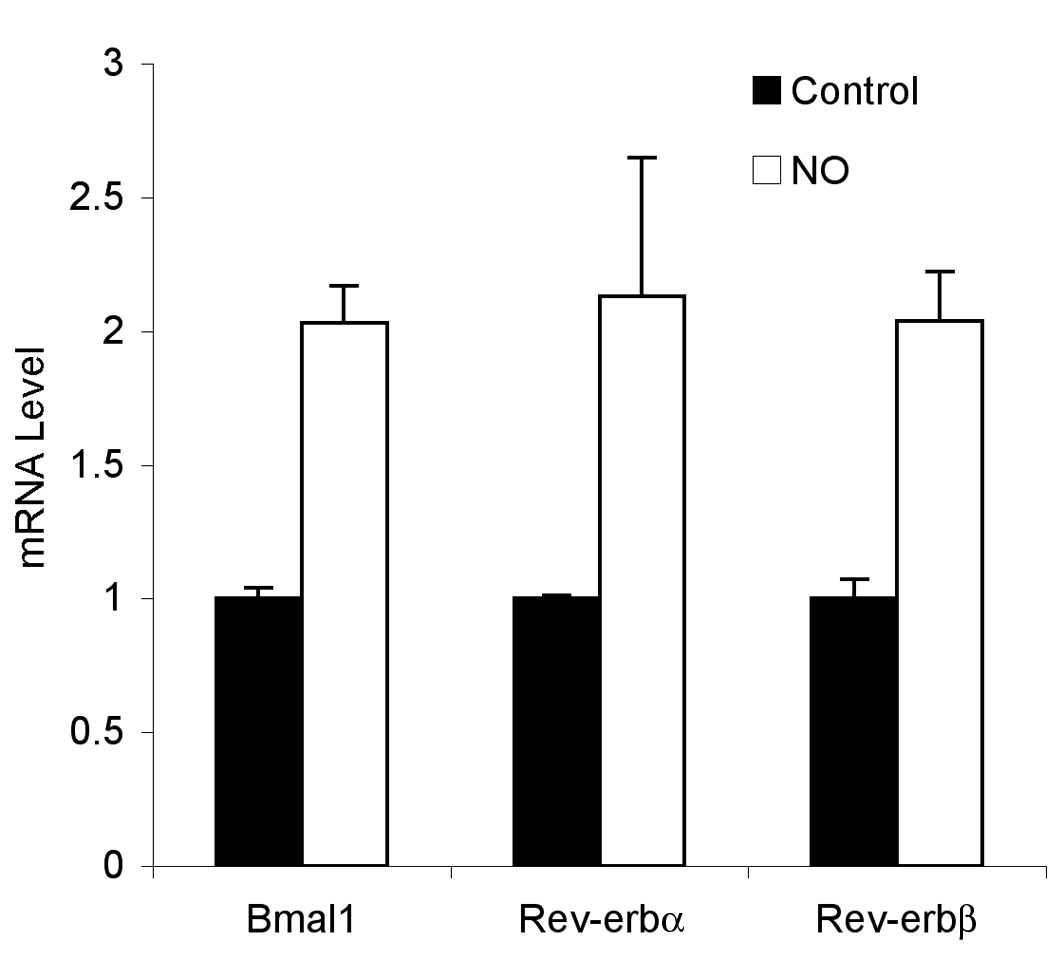

Supplement: Figure S3 — Endogenous mRNA levels of Bmal1 and Rev-erbα and β under control and 300 μM Deta/NO treatments. (53 KB TIF) [file pbio.1000043.sg003.tif]

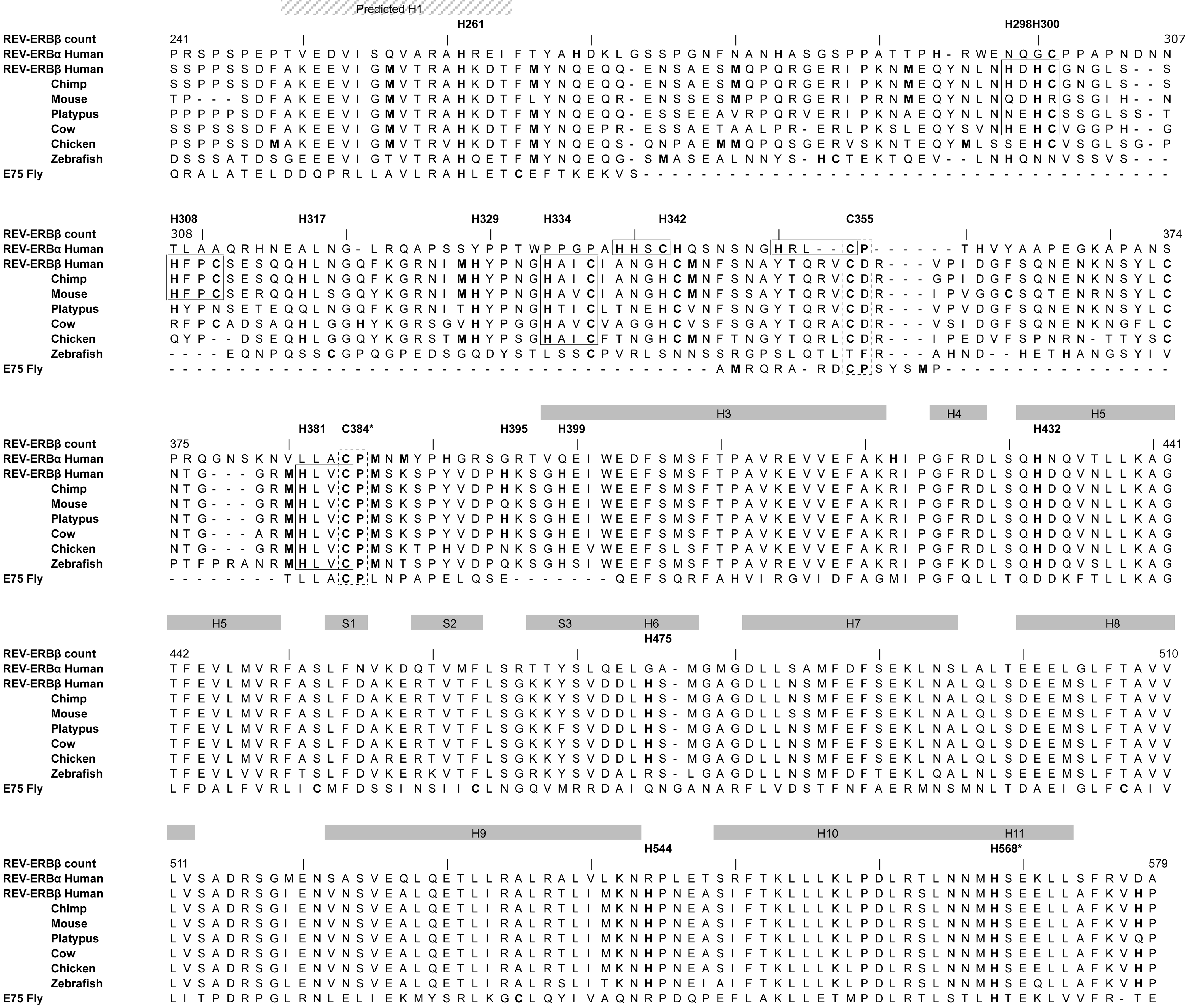

Supplement: Figure S4 — Sequences were aligned using Clustal W [106] and then adjusted manually. Determined secondary structure of heme-bound REV-ERBβ is labeled above and residues involved in heme coordination are marked (*). All residues that could coordinate heme in the loop and His, Cys through the proteins are in bold type. Dashed line, Cys/Pro motifs of putative heme responsive motifs; solid line, HXXC motifs. (177 KB TIF) [file pbio.1000043.sg004.tif]

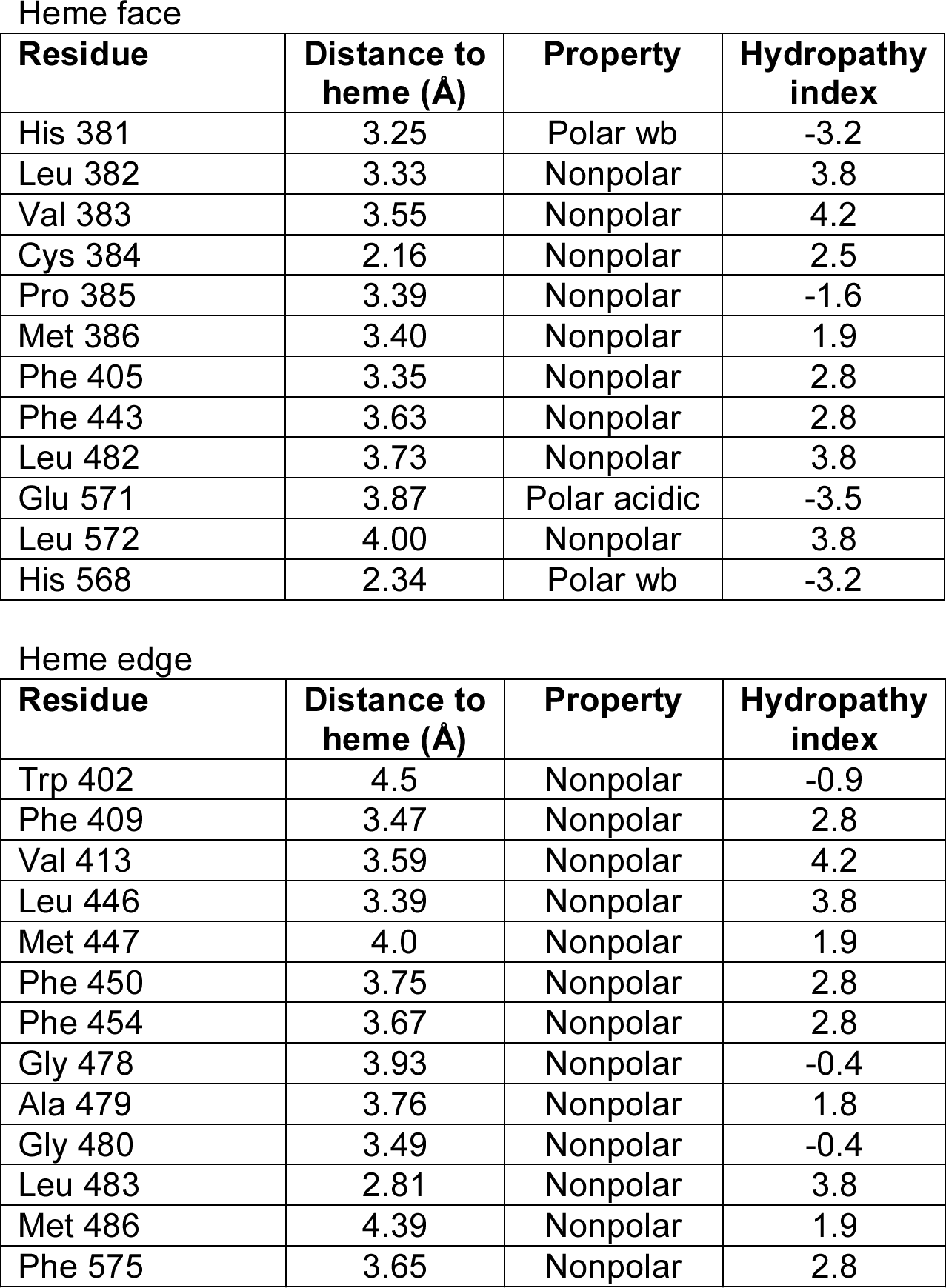

Supplement: Table S1 — Residues have been categorized based on whether they interaction with the heme face or heme edge. The table lists position, distance from heme, chemical property, and hydropathy index for each residue [107]. (487 KB TIF) [file pbio.1000043.st001.tif]
