# Supplementary material for: The Structural Basis of Gas-Responsive Transcription by the Human Nuclear Hormone Receptor REV-ERBβ
Source: PLoS Biol. 2009 Feb 24;7(2):e1000043. doi: 10.1371/journal.pbio.1000043 (PMC2652392; doi:10.1371/journal.pbio.1000043)
Supplement: Table S2 — (38 KB DOC) [file pbio.1000043.st002.doc]

**Table 1. Data processing and refinement statistics**

| **Measurement** |  |
| --- | --- |
|  |  |
| **Data processing** |  |
| Space group | P 21212 |
| Resolution, Å | 50.0–1.9 |
| No. of measured reflections | 186,684 |
| No. of independent reflections | 20,061 |
| R factor, % (overall/outer shell) | 7.5/37.4 |
| I/σ, overall/outer shell | 40.2/5.3 |
| Completeness, % | 99.6/100.0 |
|  |  |
| **Structure refinement** |  |
| Resolution, Å | 50.0-1.9 |
| No. of reflections (working set/test set) | 19008/1020 (99.6%) |
| R/Rfree, % | 19.7/23.6 |
|  |  |
| **Number of nonhydrogen atoms** |  |
| Protein | 1571 |
| Heme | 43 |
| Water molecules | 166 |
| Model rms deviation from ideality bonds/angles, Å/° | 0.014/1.41 |
